# Imtidad: A Reference Architecture and a Case Study on Developing Distributed AI Services for Skin Disease Diagnosis over Cloud, Fog and Edge

**DOI:** 10.3390/s22051854

**Published:** 2022-02-26

**Authors:** Nourah Janbi, Rashid Mehmood, Iyad Katib, Aiiad Albeshri, Juan M. Corchado, Tan Yigitcanlar

**Affiliations:** 1 Department of Computer Science, Faculty of Computing and Information Technology, King Abdulaziz University, Jeddah 21589, Saudi Arabia; njanbi0006@stu.kau.edu.sa (N.J.); iakatib@kau.edu.sa (I.K.); aaalbeshri@kau.edu.sa (A.A.); 2 High Performance Computing Center, King Abdulaziz University, Jeddah 21589, Saudi Arabia; 3 Bisite Research Group, University of Salamanca, 37007 Salamanca, Spain; corchado@usal.es; 4 Air Institute, IoT Digital Innovation Hub, 37188 Salamanca, Spain; 5 Department of Electronics, Information and Communication, Faculty of Engineering, Osaka Institute of Technology, Osaka 535-8585, Japan; 6 School of Architecture and Built Environment, Queensland University of Technology, 2 George Street, Brisbane, QLD 4000, Australia; tan.yigitcanlar@qut.edu.au

**Keywords:** tiny AI, tiny ML, distributed AI as a service (DAIaaS), fog computing, edge computing, cloud computing, skin disease diagnosis, healthcare, smart societies, smart cities, smart healthcare, reference architecture, TensorFlow

## Abstract

Several factors are motivating the development of preventive, personalized, connected, virtual, and ubiquitous healthcare services. These factors include declining public health, increase in chronic diseases, an ageing population, rising healthcare costs, the need to bring intelligence near the user for privacy, security, performance, and costs reasons, as well as COVID-19. Motivated by these drivers, this paper proposes, implements, and evaluates a reference architecture called Imtidad that provides Distributed Artificial Intelligence (AI) as a Service (DAIaaS) over cloud, fog, and edge using a service catalog case study containing 22 AI skin disease diagnosis services. These services belong to four service classes that are distinguished based on software platforms (containerized gRPC, gRPC, Android, and Android Nearby) and are executed on a range of hardware platforms (Google Cloud, HP Pavilion Laptop, NVIDIA Jetson nano, Raspberry Pi Model B, Samsung Galaxy S9, and Samsung Galaxy Note 4) and four network types (Fiber, Cellular, Wi-Fi, and Bluetooth). The AI models for the diagnosis include two standard Deep Neural Networks and two Tiny AI deep models to enable their execution at the edge, trained and tested using 10,015 real-life dermatoscopic images. The services are evaluated using several benchmarks including model service value, response time, energy consumption, and network transfer time. A DL service on a local smartphone provides the best service in terms of both energy and speed, followed by a Raspberry Pi edge device and a laptop in fog. The services are designed to enable different use cases, such as patient diagnosis at home or sending diagnosis requests to travelling medical professionals through a fog device or cloud. This is the pioneering work that provides a reference architecture and such a detailed implementation and treatment of DAIaaS services, and is also expected to have an extensive impact on developing smart distributed service infrastructures for healthcare and other sectors.

## 1. Introduction

Smart cities and societies are at the vanguard of driving digital transformation [1,2,3,4,5]. The digital transformation process involves developing digital services and systems that allow us to sense, analyze, and act on our environment with the optimality of our objectives [6,7]. Various industrial sectors are undergoing this transformation and healthcare is among the most critical sectors in need of this [8]. Several drivers are motivating the need to transform healthcare and develop preventive, personalized, connected, virtual, and everywhere healthcare services and systems [9,10,11,12]. These drivers include, among others, declining public health (due to processed food, lifestyles, etc.), increase in chronic diseases (e.g., hypertension, diabetes, heart disease), ageing population, decreasing quality of healthcare, and rising healthcare costs for the public and governments [6]. Due to the restrictions placed because of COVID-19, the difficulty of accessing to public healthcare has aggravated and amplified the need for virtual and everywhere healthcare [4].

The technology-related drivers to provide distributed services include the need to bring intelligence near the user (at the fog and edge layers) for reasons such as privacy, security, performance, and costs [13,14,15,16,17,18]. These drivers are not specific to healthcare alone and are driving all of the sectors in which data is generated at the edge and/or in which decisions need to be made instantaneously and intelligently by the user at the edge [19,20,21,22,23].

Motivated by these drivers, this paper proposes, implements, and evaluates a reference (software) architecture called Imtidad that provides distributed Artificial Intelligence (AI) as a Service (DAIaaS) over the cloud, fog, and edge layers using a case study of a service catalog with 22 Deep Learning-based skin disease diagnosis services. These services belong to four service classes that are distinguished by software platforms (containerized gRPC, gRPC, Android, and Android Nearby) and are executed on a range of hardware platforms (Google Cloud, HP Pavilion Laptop, NVIDIA Jetson nano, Raspberry Pi Model B, Samsung Galaxy S9, and Samsung Galaxy Note 4) and four network types (Fiber, Cellular, Wi-Fi, and Bluetooth). A selection of four AI models are provided for the diagnosis; two of these are standard Deep Neural Networks, and the other two are Tiny AI versions to enable their execution on smaller devices at the edge. The models have been trained and tested on the HAM10000 dataset containing 10,015 dermatoscopic images.

The services have been evaluated against several benchmark criteria, including model service value, processing time, response time, data transfer rate, energy consumption, and network transfer time. The service values have been computed and compared in terms of their speed and energy consumption. A Deep Learning (DL) service on a local smartphone provides the best service in terms of energy, followed by a Raspberry Pi edge device. A DL service on a local smartphone provides the best service (also in terms of speed), followed by a laptop device in the fog layer.

Imtidad is an Arabic word indicating the “extending” or “extension” (to the cloud, fog, and edge) nature of our reference architecture. The services are being extended in both directions, from cloud to fog and edge, and from edge to fog and cloud.

**Figure 1:**
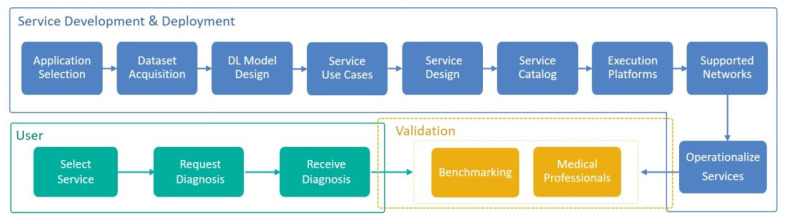
The Imtidad reference architecture (a high-level view).

To help the reader conceptualize the proposed work, Figure 1 provides a high-level view of the Imtidad reference architecture. The reference architecture is described at length in this paper. The three perspectives of the reference architecture are: the service development and deployment perspective, the user view perspective, and the validation perspective. The service development and deployment perspective provides guidelines on developing and operationalizing the services: an application such as skin lesion diagnosis is selected for the provision of related distributed services followed by acquiring the necessary data, AI model designs, service use cases, service design, composing these into a service catalog, porting these to the execution platforms and networks, operationalizing the services, evaluating and validating them against benchmark criteria, medical professionals, and other sources of knowledge. The user view perspective includes selecting and requesting a service from the service catalog, receiving the diagnosis, and validating it. The validation perspective is shared with both the user view and the service designers and providers view because it is meant to allow all of them, as well as third parties, such as auditors, to validate. A more detailed view of the reference architecture is provided in Section 3 and Section 4.

The contributions of this paper can be outlined as follows:

- This is the first paper in which a reference architecture for distributed AI-as-a-service is proposed and implemented; a healthcare application (skin lesion diagnosis) is developed and studied in great detail, with a catalog containing several AI and Tiny AI services supported on multiple software, hardware, and networking platforms; and several use cases are evaluated using multiple benchmarks.
- The services are designed considering innovative use cases, such as a patient at home taking images of their skin lesion and performing the diagnosis by themselves with the help of a service or a travelling medical professional requesting a diagnosis from a fog device or cloud. The users of the services provided by this architecture can be patients, medical professionals, the patients’ family members, or any other stakeholder. Similarly, the services can be used by someone who has the disease diagnosis model, or the image, or both, since the resource (image or model) may be requested from other providers.
- The proposed work is highly novel and is expected to produce high impact due to the developed reference architecture; the service catalog offering a large number of services; the potential for the implementation of innovative use cases through the edge, fog, and cloud as well as their evaluation on many software, hardware, and networking platforms; and a detailed description of the architecture and case study.
- The existing works on distributed AI either focus on distributed AI methodologies [24,25] or distributed applications development [26,27,28], or application migration to fog and edge [29,30]. In contrast, this paper broadly aims to provide theoretical and applied contributions on decoupling application development from AI by using the distributed AI as a Service (DAIaaS) concept to coordinate, standardize, and streamline existing research on distributed AI and application migration to the edge. The decoupling of application development from AI is needed because it allows application, sensor, and IoT developers to focus on the various domain-specific details, relieve them from worries related to the how-to of distributed training and inference, and help systemize and mass-produce technologies for smarter environments. The Imtidad reference architecture and case study, given in this paper, outlines the whole process and roadmap of developing a service catalog using distributed AI as a Service, and, essentially, this provides a blueprint and procedure for decoupling applications and AI, enabling smart application development as a foundation for smarter societies. The approach allows development of unified interfaces to facilitate both independent and collaborative software development across different application domains. This is a continuation of our earlier research, where a DAIaaS concept was proposed and investigated using simulations [13].

The rest of the paper is organized as follows. Section 2 reviews the related works. Section 3 describes the reference architecture, methodology, and service catalog. Section 4 details the system architecture and design for the skin disease diagnosis case study. Section 5 provides results and their analysis. Section 6 concludes the paper and provides future lines of research.

## 2. Related Works

This section reviews the literature related to topics of this paper, distributed AI for skin diseases diagnosis over the edge. Section 2.1 discusses the works related to distributed artificial intelligence over cloud, fog, and edge. Section 2.2 reviews the works related to skin disease diagnosis using AI and Section 2.3 discusses the research gap.

### 2.1. Distributed Artificial Intelligence (DAI) over Cloud, Fog, and Edge

Distributed Artificial Intelligence (DAI) allows AI to be distributed across multiple agents, processes, cores, physical, or virtual, computational nodes with the aim of sharing data, improving data processing capabilities, and providing faster, privacy-preserved, node-local, global, or system-wide solutions [13]. Distributed AI on clouds has been the focus of many proposals, [31,32], for intensive computation or global knowledge sharing. Edge Intelligence (EdgeAI) and fog intelligence are among the main DAI approaches where AI models are distributed across fog nodes (intermediate nodes between edge and cloud layers) or network edges [33]. Models can be pre-trained on powerful machines (cloud), then modified and optimized to run in the resource-constrained edges. Edges, fogs, and cloud can also collaborate where some of the pre-processing and less-intensive computations are performed in edges and global processing performed in the cloud [13].

Several research studies have discussed the convergence of edge, fog, and AI, as well as their various architectures [30,34,35,36,37,38,39,40]. Pattnaik et al. [41] have proposed and evaluated different approaches to distribute ML across cloud and edge layers, including a variety of distributed edge and cloud-based training and inference with either local or global knowledge. Muhammed et al. [33] proposed UbiPriSEQ, a framework to optimize privacy, security, energy efficiency, and quality of service (QoS). UbiPriSEQ uses Deep Reinforcement Learning to optimize local processing and offloading on edge, fog, and cloud. Sparse matrix-vector multiplication (SpMV) is used as an application to implement and evaluate the proposed framework UbiPriSEQ. In our earlier work, Janbi et al. [13], we proposed a DAIaaS framework to standardize distributed AI provisioning across all layers (edge, fog, and cloud) aiming to facilitate the process of generic software development across different application domains and allow for developers to focus on the domain-specific details rather than how-to develop and deploy distributed AI. To this end, multiple case studies and several scenarios, applications, distributed AI delivery models, sensing modules, and software modules were developed to explore various architectures and understand performance barriers.

Another recently emerging direction is Federated Learning (FL), where edge devices collaborate to train ML models. Model aggregation can be performed centrally in the cloud or be distributed between nodes. Gao et al. [42] have proposed a cloud-edge collaborative learning framework with an elastic local update method. In addition, the n-soft synchronization approach has been proposed that combines both synchronous and asynchronous approaches. Chen et al. [43] have proposed a federated transfer learning approach for healthcare wearables to train global models across different organizations securely. Fully decentralized FL approaches, where no central server and models are aggregated directly by edge devices, have also been proposed in the literature. Hegedűs et al. [44] have provided a comparison of central FL and decentralized FL as well as introduced two optimization techniques for decentralized FL, a token-based flow control and partitioned models subsampling. Kim et al. [45] have proposed an architecture of FL based on blockchain technology to enable secure local model exchange. Both verification and rewards systems are designed to support the exchange process between edges. The existing works on federated learning have focused on federated training over distributed devices, while our work differs from it and complements it, both in the broad aims of our research and the specific contributions of this paper (as highlighted in Section 2.3 (Research Gap) and elsewhere in the paper).

#### 2.1.1. Tiny AI and Edge: Research and Frameworks

Table 1 gives a summary of research papers that utilized Tiny AI models, i.e., lighter versions of AI models on edge devices. Tiny AI models are customized AI models that are optimized or compressed to minimize the requirements for model memory and computation power. All the listed research has used TensorFlow Lite [46] to optimize and deploy the AI models locally. For each research in the table, the application domain, the specific application under that domain, and the adopted AI model are specified. Zebin et al. [47] have designed and implemented a tiny CNN model to optimally monitor human activity recognition using mobile devices. In the domain of the autonomous vehicles, a traffic sign recognition Tiny DL model based on Single Shot MultiBox Detector (SSD) has been developed by Benhamida et al. [48]. Alsing [49] has evaluated different tiny AI models for note detections in a smart home environment. For the security domain, Zeroual et al. [50] have developed a face recognition authentication model on mobile devices to authenticate users before accessing cloud services. Alternatively, Ahmadi et al. [51] have proposed an intelligent local malware detection approach for android devices based on random forests classifier. Soltani et al. [52] have developed a Tiny Deep CNN model for Signal Modulation Classification that identifies signals SNR region for wireless networks. A Tiny AI model on Unmanned Aerial Vehicles (UAV) has been proposed by Domozi et al. [53] to detect objects in search and rescue missions.

Regarding the deployment of AI at the edges, a few frameworks have been proposed and developed to run AI models on edge devices. These include Caffe2 [54], TensorFlow Lite [46], and PyTorch Mobile [55]. These frameworks support various edge platforms such as Android, iOS, and Linux and customize AI models to fit within the resource-constrained edge.

#### 2.1.2. Distributed AI in Healthcare

EdgeAI is still in its infancy and attracting more researchers and companies to bring AI closer to users [34]. It aims to provide distributed, low-latency, reliable, scalable, and private AI services [35]. Many applications that require real-time responses can utilize edgeAI, such as autonomous vehicles, smart homes, smart cities, and security [47,48,49,50,51,52,53]. There are some works that have considered distributed AI for healthcare, which is the focus of this work too. Zebin et al. [47] have proposed a human activity recognition framework to run on mobile devices. They used batch normalization for CNN recognition tasks using data from wearable sensors. Isakov et al. [31] have developed a monitoring and detection system that aims to detect falls accurately through the use of mobile devices. The mobile devices are used for preprocessing and they perform a non-linear analysis on the cloud. Hassan et al. [32] proposed a remote pain monitoring system based on a fog-based architecture to process patient biopotential signals locally and detect pain in a real-time manner. They offloaded some of the processing to the cloud in case of local resource shortage and provided remote access through a web application. Muhammed et al. [56] have addressed the challenges of meeting network quality of service (QoS) requirements including network latency, bandwidth, and reliability challenges for delivering real-time mobile healthcare services.

### 2.2. Skin Lesion Diagnosis

Health information technology systems such as clinical decision support (CDS) systems are designed to support physicians and other health professionals in their decision-making tasks. AI based Computer-Aided Diagnosis (CAD) systems have been subject to rapidly growing interest for the diagnosis of skin disease [57]. They are used as a “second opinion” tool that assists radiologists and physicians in image interpretations and diseases diagnosis. There has been a continuous increase in skin cancer cases rates around the world, so, given that it is the most common cancer in the United States and worldwide [58], more research must be done in this area. Especially, since an accurate and early diagnosis of skin cancer would improve treatment and survival rates [59]. Computer vision algorithms are used to analyze images and identify abnormal structures. This helps professionals to detect the earliest signs of abnormality and support their evaluation. Clinical imaging and dermatoscopy are now considered to be an essential part of the dermatology clinics for diagnosis, treatment, follow-up, and documentation [60,61]. Skin diagnosis (and identifying benign and malignant skin lesions) is an important factor in the early detection and prevention of skin cancer. Automated skin diagnosis using dermoscopy and AI might also let patients avoid skin biopsy [62]. DL is one of the AI approaches that are becoming very popular for dermoscopic images classification problem. This has been boosted by the introduction of many dermoscopic datasets that are publicly available [57]. These datasets consist of labeled images belonging to various types of benign and cancerous skin lesions. Training DL model with such datasets would create an appropriate and accurate model for CAD systems.

Several research studies have been proposed in the literature aiming to improve the accuracy of skin diagnosis [63,64,65,66,67,68,69,70,71,72]. Convolutional neural networks (CNN) are adopted in most proposals [63,64,65,66,67,68,69,70,71], except in [72] where the authors proposed fuzzy classification for skin lesion segmentation. Some proposals have considered other information or data in the diagnosis process such as demographic and medical history [66] and sonification (audio) [73]. Pretrained CNN models have been retrained and evaluated in [63,65,66,67,68,69,71] and multiple CNN models have been ensembled in [64,66,69,70,73]. A review of DL segmentation, classification, and pre-processing techniques for skin lesion detection is provided in [74]. Table 2 summarizes the literature that has been reviewed in this subsection, related to skin disease diagnosis.

### 2.3. Research Gap

The literature review presented in this section has evidenced the current research gap with no earlier reference architectures on DAIaaS and no implementations of skin disease diagnosis on fog and edge. This is the first research where a reference architecture for DAIaaS is proposed and implemented, and a healthcare disease diagnosis service is developed and studied in great detail, with a catalog containing several AI and Tiny AI services supported on multiple software, hardware, and networking platforms, as well as several use cases evaluated using multiple benchmarks. The services are designed to enable different use cases such as a patient at home taking images of their skin lesion and performing the diagnosis by themself with the help of a service, or a travelling medical professional requesting a diagnosis from a fog device or cloud. The users of the service can be patients, medical professionals, the family members of the patient, or any other stakeholder. Similarly, the services can be used by someone who has the disease diagnosis model, or the image, or both, by requesting the required resource (image or the model) from other providers. The novelty and high impact of this research lies in the developed reference architecture, the service catalog offering many services, the potential for the implementation of innovative use cases through the edge, fog, and cloud, and their evaluation on many software, hardware, and networking platforms, as well as a detailed description of the architecture and case study.

Commenting on the specific application we have selected for this paper, i.e., skin disease diagnosis (this comment applies to similar applications), it is important to note that having an accurate disease diagnosis model is not enough; the deployment of the model for real-time usage is an essential part of the AI system development. This includes where and how the model is going to be installed. First, both model size and complexity will influence the processing or inference time, especially with resource constrained devices. In addition, the emerging trend of virtual and mobile services including healthcare services, which are required as a result of the current COVID-19 pandemic, will require innovative and flexible architectures to support them. Therefore, the development of quick and accurate diagnosis methods for physicians must intrinsically consider in their designs the distributed architectures that these diagnosis methods will be deployed on.

## 3. Imtidad Reference Architecture, Methodology, and Service Catalog

This section describes our proposed Imtidad reference architecture for creating distributed AI services over the cloud, fog, and edge layers and describes the service catalog, service use cases, and the service evaluation benchmarks. The section is organized as follows. The reference architecture overview is provided and elaborated in Section 3.1. A series of use cases (e.g., a user takes a photo of a lesion on their skin and instantaneously attempts to diagnose it using their preferred service from the service catalog) are outlined in Section 3.2. An implementation of the reference architecture using a service catalog, designed as part of this research, is described in Section 3.3. A description of execution platforms is provided in Section 3.4. The metrics that have been used to evaluate and compare the services are defined and explained (service energy consumption and service values) in Section 3.5.

### 3.1. Reference Architecture and Methodology Overview

**Figure 2:**
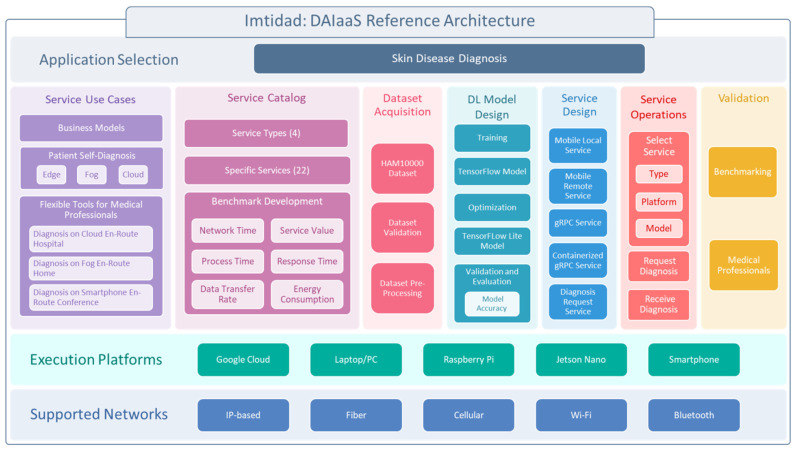
Imtidad reference architecture.

The Imtidad reference architecture is proposed as a blueprint and procedure for decoupling applications and AI and streamlining the design and deployment of distributed AI services over the cloud, fog, and edge layers. Figure 2 depicts the Imtidad reference architecture for the skin disease diagnosis case study. The figure can be considered an insanitation or refinement of the Imtidad reference architecture for a given application; in this case skin disease diagnosis. The architecture lists all required services to create new DAIaaS services from the selection of the application to service production and operations. Each of the rectangular blocks (e.g., Service Design) in the figure can be considered a component or a service, and these services can independently and asynchronously talk to each other to create services and service catalogs.

**Figure 3:**
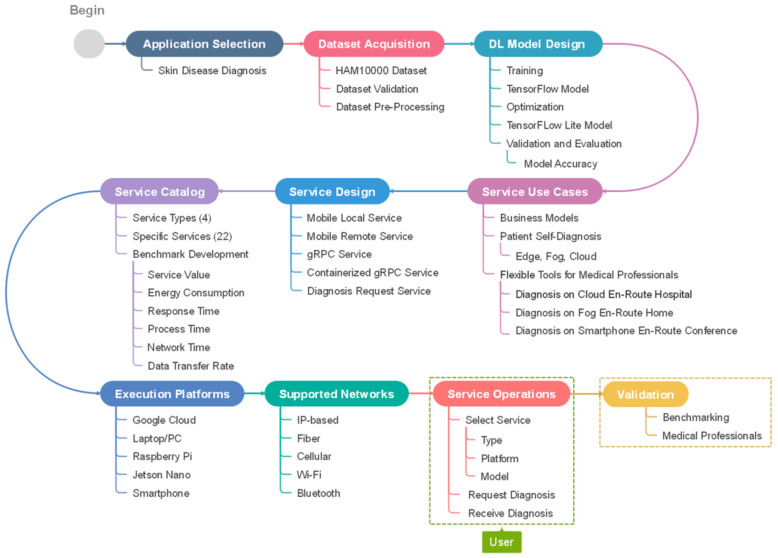
Workflow Diagram for creating a skin disease diagnosis catalog refined from Imtidad Reference Architecture.

Figure 3 depicts a sequential workflow diagram for creating a skin disease diagnosis catalog. It is created by refining Imtidad Reference Architecture. The service development and deployment process begins with a selection of an application domain, in this case, skin disease diagnosis. A dataset is required for the selected application, so that the designed model may be trained and validated. The dataset acquisition process includes dataset validation and pre-processing in preparation for training. Then, Deep Learning models are designed, trained, optimized, and validated. First, the TensorFlow (TF) model is generated, then, an optimized version is created, which, in this case, was the TensorFlow Lite (TFLite) model. Use cases are determined considering possible scenarios and business models. After that, different types of services may be designed to provide support in a series of scenarios. A service catalog is created to communicate and present various service models to users (see Table 3 and Section 3.2 for details). In addition, service providers need to find a way to benchmark services by developing evaluation metrics such as service values, energy consumption, and response time. Several execution platforms and networks are selected, and the designed services are deployed. When the services are ready for operation, the users can choose one of the services from the catalog and send their diagnosis request. External opinion might be required for validation, in this case healthcare professional opinion can be used to validate the predicted diagnosis. Validation can be done by users, service designers and providers, or a third party such as auditors.

### 3.2. Service Use Cases

Use cases are identified considering possible scenarios and business models for provisioning distributed AI services and skin disease diagnosis services, over the cloud, fog, and edge layers. These have been used to design a variety of services that suit different conditions and requirements. Services are listed in a services catalog for the user to select one of them and use it to diagnose a lesion image. The design of skin disease diagnosis services involves and concerns all parties including patients, patients’ families, medical professionals, and, even, service providers. Patients and medical professionals are the direct users of the system and they are looking for instantaneous results and services available all the time and everywhere, while service providers aim for users’ satisfaction by providing high QoS and at the same time protecting their product and copyrights.

Local services in smartphones, where model and image classification tasks are performed locally in the user device, guarantee a real-time response with no requirement for an Internet connection, and will preserve the user’s privacy as the images stay on the user’s device. This kind of service can be used by patients or doctors anywhere using their own smartphones. However, this will only work if the user’s device has the required resources needed to store and run AI models, and model accuracy may be compromised when converted into the Tiny version. On the other hand, remote services in smartphones, would extend the service capability and enable collaboration between edge devices. Services from nearby devices can be used when the users’ devices are either unable to process the image locally or they are looking for more accurate results. In this case, users can collaborate and provide services to each other without having to share their models. In addition, the DL model service providers may also want to keep their model’s copyrights and not share them, and at the same time, they want to guarantee service availability. To accomplish that, the service provider can provide a secure device (smartphone) in the facility (e.g., clinic) or with the medical professional to carry anywhere. In this case, skin images will be sent to the local device in the local network but not through the internet, which will provide some level of privacy for the users.

Mobile devices (smartphones) are limited in their capabilities, therefore, devices such as laptops, NVIDIA Jetson nano, and Raspberry Pi can be used in edge or fog layers to run more complicated models or serve a large number of users simultaneously. These devices can be provided by service providers can and placed in hospitals, clinics, or, even, homes, to serve medical professionals and other users. Devices at the edge or fog layers would increase service availability and the level of user privacy and security. Nevertheless, they are incomparable with the cloud where resources are almost unlimited. The cloud is the original service provisioning platform for AI applications though services provided from the cloud have a higher latency and more congested networks. Services at the cloud can be used in case other local services at edge or fog layers are busy or absent. Moreover, DL model service can be resides in the cloud, and data or local models can be uploaded to it for model retraining to improve the global model accuracy.

### 3.3. Service Catalog

The service catalog lists all diagnosis services with their characteristics for the users to choose from. Diagnosis services are responsible for image classification. A total of 22 services are produced from a combination of various types of services, devices, and models (see Table 3) that suit different purposes. For each service, the service type, layers, devices, network, and models are listed. There are four different skin disease diagnosis service types, namely, local mobile service, remote mobile service, gRPC service, and containerized gRPC service. These services can be run on different layers of the network architecture including cloud, fog, and edge. Seven different devices are used for evaluation that varies in their capabilities. Google cloud virtual machines (VMs), a laptop, an NVIDIA Jetson nano, two Raspberry pi (4G and 8G), and two mobile devices (Samsung Galaxy S9 and Samsung Galaxy Note 4). Wi-Fi local area network (LAN) and the Internet wide area network (WAN) are both considered, including fiber and cellular networks. An Internet connection is required for cloud communications, but all other levels are deployed in the local network which means that their traffics is going through a Wi-Fi modem. Nevertheless, they may be deployed farther than this on a base station on other LANs close to the user. The four developed models (A, ALite, B, and BLite) are considered for all devices, though only ALite and BLite are possible for some devices due to device capability limitations. This service catalog is designed for our specific case study to show a practical example of service catalogs. This means that all sorts of devices and networks could be used to design the user’s services, and they are not limited to what is specified here. Table 4 lists the acronyms and their definitions that have been used use throughout the paper for the 22 services in the service catalog.

### 3.4. Devices and Hardware Platforms

**Figure 4:**
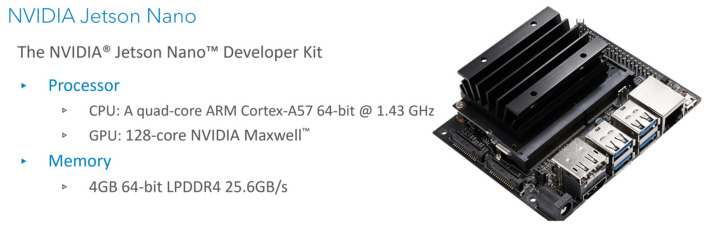
NVIDIA Jetson Nano.

**Figure 5:**
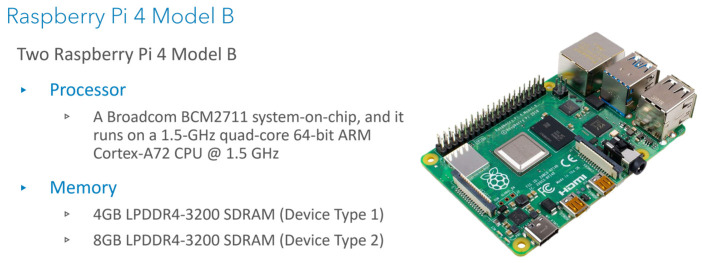
Raspberry Pi 4 Model B.

**Figure 6:**
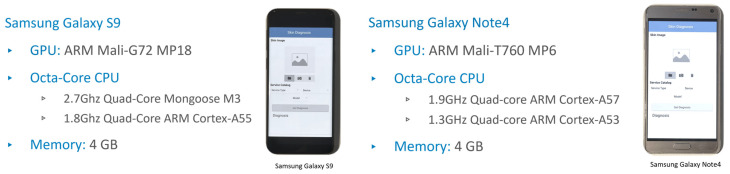
Samsung Galaxy Smartphones.

Seven different execution platforms are adopted in the service catalog. Google Cloud Run is selected for the cloud services which is a serverless platform that facilitates running invocable Docker container images via requests or events. Services are the main resources of the Cloud Run and each has a unique and permanent URL. Services are created by deploying a container image on infrastructure that is fully managed and optimized by Google. Service configuration includes maximum allocated memory limit, number of assigned virtual CPUs (vCPUs), and maximum number of requests (concurrent requests). An HP Pavilion laptop has been used as the fog node in our experiments. It comprises an Intel^®^ Core™ i7-8550U CPU and 8 GB Memory. The CPU has a total of 4 cores and 8 threads with a base frequency of 1.80 Ghz and a maximum single-core turbo frequency of 4.00 Ghz. Two types of single-board computers have been used NVIDIA Jetson nano and Raspberry Pi. NVIDIA Jetson nano is a platform designed by NVIDIA to run AI applications at the edge. The used Jetson Developer Kit is equipped with 128-core NVIDIA Maxwell™ architecture-based GPU, Quad-core ARM^®^ A57, and 4 GB 64-bit Memory. Figure 4 gives a brief of Jetson nano specifications and a picture of the device. Raspberry Pi is a tiny and low-cost single-board computer. Several generations of Raspberry Pi have been released during the years. In this research, two Raspberry Pi 4 Model Bs have been used. Both cards have the same Quad-core ARM Cortex-A72 processor, but one has 4 GB memory and the other has 8 GB memory. Figure 5 gives a brief of Raspberry Pi specifications and a picture of the device. Two Samsung smartphones have been used, Galaxy S9 and Galaxy Note 4. Samsung Galaxy S9 comes with ARM Mali-G72 GPU and Octa-Core CPU (Quad-Core Mongoose M3 and Quad-Core ARM Cortex-A55), Samsung Galaxy Note 4 comes with ARM Mali-T760 GPU and Octa-Core CPU (Quad-core ARM Cortex-A57 and Quad-core ARM Cortex-A53), and both have 4 GB memory. Figure 6 gives a brief of the smartphone’s specifications and provides pictures for both smartphones. A full depiction of the Imtidad testbed is given in Section 4.

These platforms can be located in different layers at cloud, fog, or edge. The main difference between these layers is the place where processing occurs. The cloud is located far away from the users on datacenter/s and accessed through an Internet connection, Wide Area Network (WAN). On the other hand, fog is located near users and the edge, on the same Local Area Network (LAN) or a near LAN, and it does not require an Internet connection. Fog devices might be located in streets, base stations, houses, cafes, hospitals, etc., to serve local users, while the cloud is designed to serve a large number of users. The cloud provides resources on-demand and can scale up easily. Though cloud and fog might have the same type of CPUs, cloud can increase the number of located CPUs on request or with high demands while fog resources are limited. In our case study, the cloud is the Google datacenter, specifically the Google Cloud Run platform. For the Containerized gRPC Service, two CPUs are allocated with an 8 GB memory limit and 80 concurrent requests at a time. The Fog is the HP Pavilion Laptop with an Intel^®^ Core™ i7-8550U CPU and 8 GB Memory. Other devices on the LAN, such as NVIDIA Jetson nano and Raspberry Pi, can also be referred to as fog but for simplicity, we only refer to the laptop as Fog.

### 3.5. Service Evaluation

To provide a way to evaluate various services in the service catalog, service energy consumptions and service values have been used as evaluation metrics. The estimated service energy consumption (*e_t_*) for each task is calculated as an aggregated value of the data transfer energy consumption and the device energy consumption (Equation (1)).

(1) $$e_{t}=\left(\varepsilon_{n}*d*\;t\right)+\left(\eta *p\right)$$

The first part of Equation (1) calculates the data transfer energy consumption where *ε_n_* is the estimated energy of a gigabyte transfer on a network of type *n*. Andrae and Edler [76] energy consumption estimations of wired fixed access network, wireless access network, and Wi-Fi for 2020 have been used in the calculation. The used energy consumption averages are 0.195 kWh/GB, 0.5435 kWh/GB, and 0.12 kWh/GB for network types Fiber, 4G, and Wi-Fi, respectively. The term *d* is the size of the transferred data for each task, including both request and response packets. The term *t* is the average network time which is calculated as the difference between the response and processing time. The second part of Equation (1) calculates processing energy consumption for the service device, where η is the estimated device processing energy, which varies depending on the type of device and its specification (see Table 3 for the devices’ energy-related data). The term *p* is the average processing time for each request. The terms *d*, *t*, and *p* are all averages of data collected from the experiments.

Relative values are calculated to compare two absolute values to each other, which in return provides a better way to compare service-to-service values than the absolute values such as response time, process time, energy consumption, etc. Two relative values are computed service energy value (eValue) and service speed value (sValue), as a way to benchmark different services in terms of their accuracy, energy consumption, and speed (response time). Service eValue provides accuracy-to-energy relative value, considering model accuracy and service energy consumption. Equation (2) is used to calculate the services eValue, where *e_t_* is the estimated service energy for each task using Equation (1) and *a* is the model accuracy, which represents the percentage of true disease prediction. The model accuracy is discussed in detail, for each model, in Section 4.3. Service sValue provides accuracy-to-speed relative value considering model accuracy and service response time. Service sValue is calculated using Equation (3), where *r_t_* is the average response time for each task and *a* is the model accuracy.

(2) $$\;\mathrm{eValue}=\frac{\;\;\;\;\;\;\;\;\;\;\;\;\;\;\;\;a\;\;\;\;\;\;\;\;\;\;\;\;\;\;\;}{\;e_{t}}$$

(3) $$\mathrm{sValue}=\frac{\;\;\;\;\;\;\;\;\;\;\;\;\;\;\;\;a\;\;\;\;\;\;\;\;\;\;\;\;\;\;\;}{r_{t}\;}$$

Note that the purpose of computing service value is to define a method for benchmarking services and it can be considered independent of the parametric values in the equations, such as *e_t_*, *ε_t_*, *ε_n_*, η, etc., as they can be replaced by more accurate and specific values.

## 4. System Architecture and Design (Skin Lesion Diagnosis Services)

**Figure 7:**
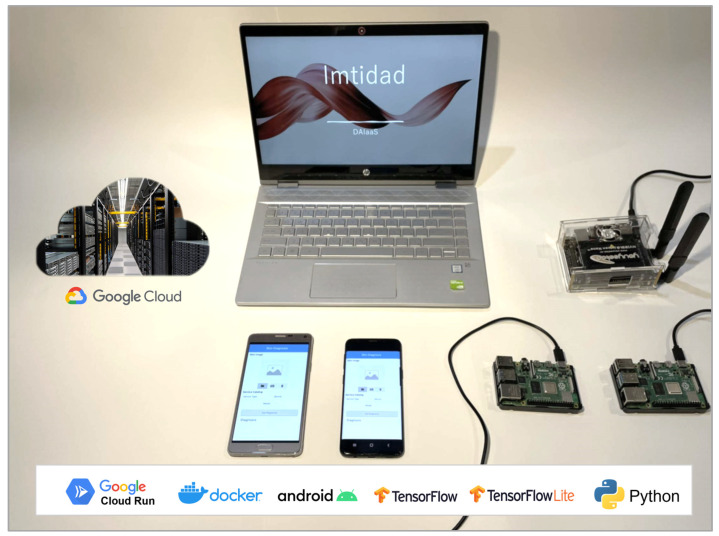
Imtidad testbed: devices and platforms.

This section describes the design of the proposed distributed skin disease diagnosis services. Figure 7 gives a depiction of Imtidad testbed including its devices and platforms both hardware and software. The testbed consists of one NVIDIA Jetson nano card, two Raspberry Pi cards, two Samsung smartphones, one HP Pavilion Laptop, and access to the Google Cloud Run platform. All these are connected through a wireless connection and equipped with the required software platforms. The white box on the bottom lists the software platforms used in the Imtidad testbed. The specifications of each device have been discussed in detail in Section 3.4, and the rest of this section will explain the whole system architecture and its components in detail.

This section is organized as follows. First, an overview of the system is provided and elaborated in Section 4.1, then each service is discussed in detail in the rest of the section. Section 4.2 discusses available skin datasets and the selected dataset for model training. The DL model service and model design and evaluation are described in Section 4.3. The following sections discuss each service as follows: Section 4.4 the mobile local service, Section 4.5 the mobile remote service, Section 4.6 the gRPC service, Section 4.7 the containerized gRPC service, and Section 4.8 the diagnosis request service.

### 4.1. System Overview

The case study presented in this paper focused on the classification of the diagnoses of common pigmented skin lesions through Deep Learning-based analysis of multi-source dermatoscopic images, to elaborate on our distributed Deep Learning DL-as-a-service reference architecture. A service catalog, containing 22 different services, has been designed and implemented to investigate the proposed Imtidad reference architecture. These services belong to four service classes (or service types) that are distinguished by their varying communication and software platforms (containerized gRPC, gRPC, Android, and Android Nearby). Android service class is referred to as “Mobile Local” and the Android Nearby service class as “Mobile Remote”. The services are executed on a range of platforms or devices (both terms are used, platforms, and devices, interchangeably according to the context) including Google Cloud (Compute Node), HP Pavilion Laptop, NVIDIA Jetson nano, Raspberry Pi Model B (8 GB), Raspberry Pi Model B (4 GB), Samsung Galaxy S9, and Samsung Galaxy Note 4. These devices could exist in one or multiple of the three distributed system layers, cloud, fog, and edge. Service performance has been evaluated on fiber, cellular, Wi-Fi, and Bluetooth networks, although the designed services are IP-based and can use any IP-based networks. The 22 distributed AI services are based on four different Deep Learning models for skin cancer diagnosis, two of these are standard Deep Learning models, called Deep Learning “Model A” and “Model B”. The other two models are the lighter versions of the Deep Learning models A and B called “ALite” and “BLite”. The lighter models are Tiny AI models created using the Google platform TensorFlow Lite. The performance of all four models has been evaluated for all the devices, except for Raspberry Pi Model B (4 GB) and the mobile devices that were unable to execute standard models (A and B) due to the device resource limitations.

**Figure 8:**
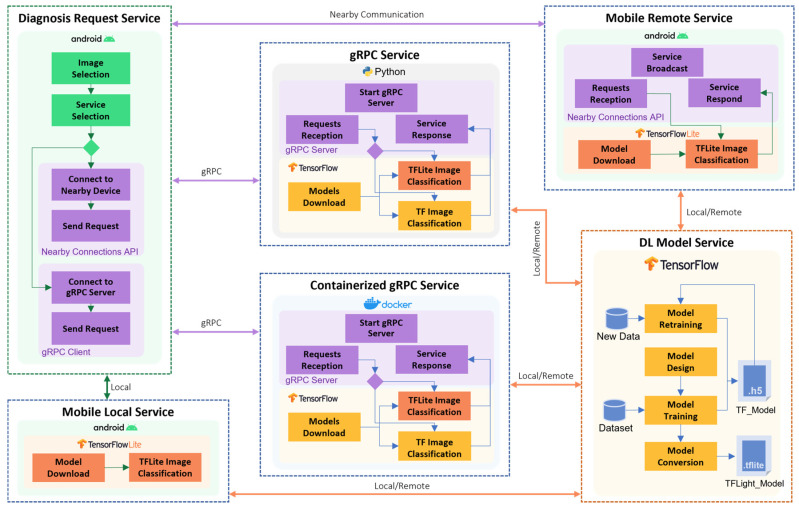
System architecture and design (skin lesion diagnosis services).

The developed system follows a service-based design architecture rather than a component-based architecture. As services are self-contained, loosely coupled, reusable, and programming language-independent components, they provide flexibility and are easy to deploy on various platforms. Figure 8 shows the system architecture, consisting of six different services: DL model service, mobile local service, mobile remote service, gRPC service, containerized gRPC service, and diagnosis request service. The arrows linking various services show the communication among them. The DL model service is responsible for designing, implementing, training, retraining, and optimizing DL models using TensorFlow. It provides two types of models: the TF_model and the TFLight_model. Four different types of services have been designed that provide skin image diagnosis (classification) services, namely, mobile local service, mobile remote service, gRPC service, and containerized gRPC service, which are explained in detail in later sections. The diagnosis request service is used by users to request skin disease diagnosis from one of the diagnosis services. The user takes or selects a skin image from their drive. Then, one of the services is selected from the provided service catalog, and a request is sent to it. Depending on the service type, a connection is established with the provider and the image is sent to the provider for classification (diagnosis). When the results are sent back, they are presented to the user.

Algorithm 1 is the master algorithm for creating new DAI services following the proposed reference architecture (see Figure 2). The algorithm comprises a list of six services that are designed and instantiated. They are shown in Figure 8, in addition to dataset acquisition and service catalog creation. The parametrization of services is used to show the instantiation of services on different devices. For instance, mobile local services are only instantiated on mobile devices while gRPC services are instantiated on various devices including PCs, laptops, Jetson Nanos, and Raspberry Pis.
**Algorithm 1:** The Master Algorithm: Create_Services(skin_disease_diagnosis)**Input**: ServiceClass skin_disease_diagnosis **Output**: service_catalog1   dataset_acquisition(skin_disease) 2   deep_learing_model_service ← design_deep_learing_model(tf_model, tf_lite_model) 3   instantiate(deep_learing_model_service) 4   service_catalog ← create_service_catalog (skin_disease_diagnosis) 5   mobile_local_service ← design_mobile_local (mobile) 6   instantiate (mobile_local_service) 7   mobile_remote_service ← design_mobile_remote (mobile) 8   instantiate (mobile_remote_service) 9   grpc_service ← design_grpc (pc, laptop, jetson, raspberry) 10  instantiate (grpc_service) 11  containerized_grpc_service ←design_container_grpc (cloud, pc, laptop) 12  instantiate (container_grpc_service) 13  diagnosis_request_service ←design_diagnosis_request (mobile, pc, laptop) 14  instantiate (diagnosis_request_service)

Algorithm 2 is a generalized algorithm for the four types of skin image diagnosis (classification) services: mobile local service, mobile remote service, gRPC service, and containerized gRPC service. It explains the service provisioning procedure followed by diagnosis services. The main function is get_diagnosis, which is called by the diagnosis request service. It takes a skin image as input and returns a list of probabilities of each class of skin disease.
**Algorithm 2**: Diagnosis_Service**Input**: skin_image **Output**: P[p_0_, …,p_C_] ∈ R   //C is the number of skin disease classes1   Function: get_diagnosis (skin_image) 2    Init: size ← model input dimension, std ← model normalization factor     //Image pre-processing 3    skin_image ← skin_image.rsize(size, size)//Resize the image to size x size 4    img_array[size, size] ← convert_to_array(skin_image)//Convert image to an array      //Normalization 5    For x in img_array 6       For y in img_array[x] 7          img_array[x, y] ← img_array[x, y]/std 8       End For 9    End For     //Classification 10    model ← load_model() //load trained model 11    P ← model.predict(img_array) 12    Return P

### 4.2. Dataset

There are several open skin datasets available. The International Skin Imaging Collaboration (ISIC) [77] has introduced many datasets from different sources as part of their annual challenge including ISBI, HAM10000, BCN_20000, and MSK Datasets. Interactive Atlas of Dermoscopy (IAD) [78] and PH2 [79] have also provided a dataset of dermoscopy images. He et al. [71] have collected two datasets, Skin-10 and Skin-100, as part of their research, but they have not been made publicly available. In this research, the HAM10000 (Human Against Machine with 10,000 training images) [80] dataset has been used to train the designed models. Table 5 lists the dataset characteristic including the number of images and classes of diagnoses. The dataset has been published in the Harvard Dataverse data repository and consists of 10,015 dermatoscopic images belonging to seven different diagnostic categories of common skin pigmented lesions. The last column in the table shows examples of dermatoscopic images that belong to different diagnosis classes.

### 4.3. DL Models Service

The DL model service is responsible for model design, training, retraining, and optimization (see Figure 8). This service may be located locally or remotely on cloud, fog, or edge devices. However, retrieving models from different layers of the network would affect the response time. New models can be retrieved on an interval basis or as the services agreement specifies and depending on the user preferences. TensorFlow, an ML open-source tool developed by Google, is used for model development. Algorithm 3 shows the procedure that this service follows to design a model. First, the TF model is designed and trained using the given dataset. Some pre-processing is performed on the dataset images including image resizing and normalization. After training, the TF model is saved in a Hierarchical data format version 5 (H5) file which stores model weights and configuration so they can be restored anytime. Then, the TF model is converted to a TensorFlow Lite (TFLite) model which is an optimized version of the TF model to run on mobile, embedded, and IoT devices. The TFLite model is saved in a file with the (.tflite) extension. The subsections that follow present a discussion on the design, training, evaluation, and conversion of the two models used in this paper.
**Algorithm 3**: DL_Models_Service**Input**: skin_image_dataset **Output**: tf_model, tf_lite_model files1   Function: design_models (skin_image_dataset) 2    Init: size ← input dimension, std ← normalization factor 3    tf_file_name ← “TF_model”, tf_lite_file_name ← “TFLite_model”     //Model designing 4    tf_model ← design_tf_model()     //Model training 5     For skin_image in skin_image_dataset        //Image pre-processing 6      skin_image ← skin_image.rsize(size, size)//Resize the image to size x size 7      img_array[size, size] ← convert_to_array(skin_image)//Convert image to an array         //Normalization 8      For x in img_array 9        For y in img_array[x] 10           img_array[x, y] ← img_array[x, y]/std 11         End For 12       End For 13       tf_model.train_model(img_array) 14    End For//end training        //save TF model 15    tf_model.save_model(tf_file_name)        //Convert to TFLite model 16    tf_lite_model ← convert_to_tflite(tf_model) 17    tf_lite_model.save_model(tf_lite_file_name)

#### 4.3.1. TensorFlow Model Design

**Figure 9:**
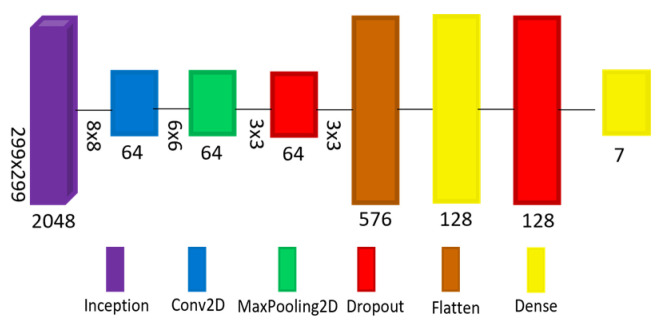
Model (A) architecture.

**Figure 10:**
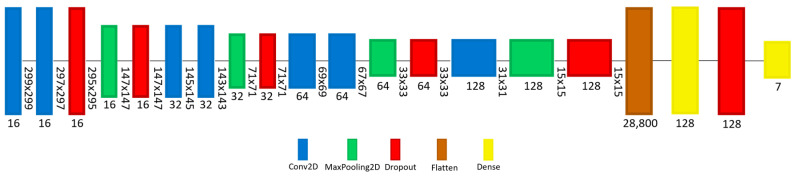
Model (B) architecture.

Two models have been designed, implemented, trained, evaluated, and converted to smaller models for edge devices. The first model (A) is based on the pre-trained model Inception v3, while the second model (B) is a pure CNN model. Figure 9 shows model (A) architecture, starting with the Inception v3 model and ending with a dense layer that has seven nodes representing each class of diagnosis. Inception v3 is a pre-trained CNN model consisting of 48 layers and trained using the ImageNet database. Multiple layers have been added to the Inception v3 model to improve its performance when it is trained with the dermatoscopic images, including 2D Convolution (Conv2D), 2D Maximum Pooling (MaxPooling2D), Dropout, Flatten, and Dense. Figure 10 shows model B architecture consisting of a series of 19 layers including 2D Convolution (Conv2D), 2D Maximum Pooling (MaxPooling2D), Dropout, Flatten, and Dense layers. The first layer, Conv2D, receives the input image of shape (299,299,3), and the last layer is a dense layer that has seven nodes representing each class of the diagnosis.

**Figure 11:**
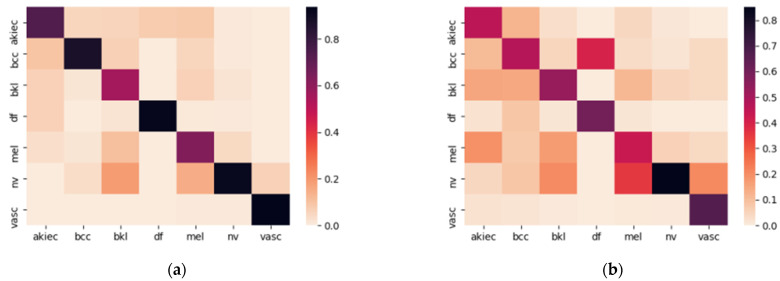
The accuracy heatmap of different classes: (**a**) Model A; (**b**) Model B.

Both models were trained using the HAM10000 dataset. The dataset was split with 60:20:20 percentages for training, validation, and testing, respectively. Model accuracy (a) was calculated for each subset of data as the percentage of true disease prediction. Model (A) had 0.96, 0.83, and 0.82 accuracies, while model (B) had 0.79, 0.78, and 0.77 accuracies for training, validation, and testing, respectively. To evaluate the accuracy of models A and B in terms of various disease classes, the heatmaps have been used to plot the confusion matrix of the test dataset predictions. Figure 11 present the heatmaps that illustrate the accuracy of classification results for the seven classes. The darker diagonal line in Figure 11a shows that Model A classification results for various classes of disease are more accurate than Model B. The nv class had the highest level of accuracy on both models and model A outperformed model B in akiec, bcc, mel, and vasc classes.

#### 4.3.2. TensorFlow Lite (TFLite) Model

After training and validating both models, TFLite Converter has been used to convert the saved TF models into TFLite models. TFLite Converter generates optimized TFLite models in a FlatBuffer serializable format identified by the (.tflite) file extension. To evaluate both models, the four model versions (A, ALite, B, and BLite) were run for the training, validation, and testing datasets. Table 6 lists the characteristic of models A and B and compares the original (TensorFlow) model and TFLite model in terms of memory footprint and accuracy. After conversion, both A and B models were reduced in size by around three-fold, with no reduction in model accuracy.

### 4.4. Mobile Local Service

In the mobile local service, both diagnosis service and diagnosis request service reside in the user device. Therefore, the user’s mobile device should have the required resources to save and run the model locally. As shown in Figure 8, the TensorFlow Lite model is provided by the DL model service in a (.tflite) format. In the diagnosis request service, the user selects a skin image and chooses the mobile local service from their catalog. The mobile local service uses the local TFLite Interpreter in the mobile device to load the model and perform image classification tasks. This type of service guarantees a real-time response and preserves user privacy as the images do not have to be sent across the network to a remote service.

### 4.5. Mobile Remote Service

**Figure 12:**
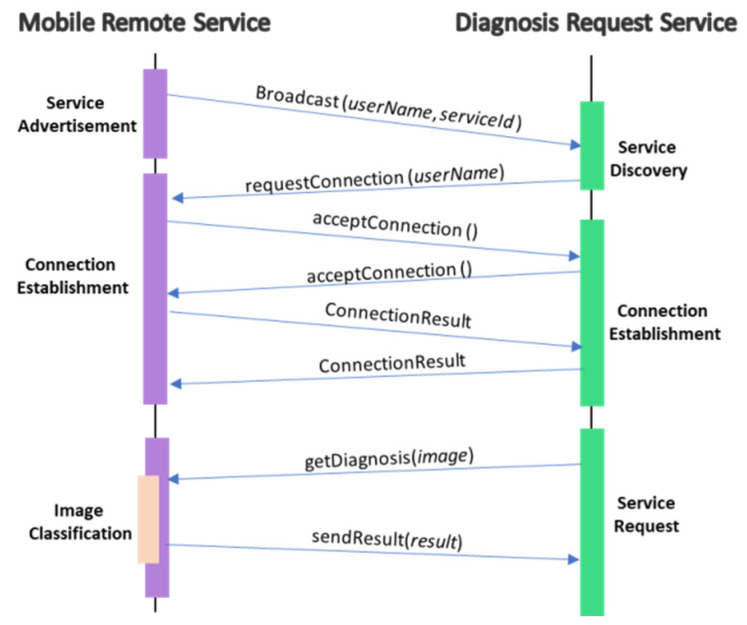
Mobile remote service nearby connection workflow.

The mobile remote service is located in mobile devices and is responsible for providing classification services to nearby devices. As shown in Figure 8, this service is equipped with a TFLite Interpreter, Android Nearby Connections API, and downloads the model from the DL model service. Android Nearby Connections API is used for service connection and management. It is a networking API provided by Android for peer-to-peer service and connection management with nearby devices using technologies such as Bluetooth, Wi-Fi, IP, and audio. This includes service advertising, discovery, connection, and data exchange in a real-time manner. Figure 12 shows messages exchanges between the mobile remote service and the diagnosis request service for the service provisioning process. The mobile remote service starts service advertisement by periodically broadcasting messages that include the service name and service ID. The diagnosis requests service listens to broadcast messages for service discovery and when the required service provider is found, the connection is requested. This invokes the connection establishment process, which includes connection acceptance from both sides and connection result acknowledgment. When the connection establishment is successful, the user can start requesting diagnosis services by sending a skin image to the provider, who uses the TFLite Interpreter to classify the image and return the result.

### 4.6. gRPC Service

The gRPC service is implemented using remote procedure calls, specifically Google Remote Procedure Call (gRPC). gRPC is a framework for building platform-independent services and providing various utilities to facilitate service implementation and deployment. Proto syntax is used to define the request and response messages that are passed between gRPC servers and clients. As shown in Figure 8, gRPC services support both TF and TFLite models for skin diagnosis. These models are provided by the DL model service. Secure Sockets Layer (SSL) protocol is used to provide secured communications between the server and client. The diagnosis request service first establishes a secure channel with the gRPC service and then sends the diagnosis request, including the skin image. When the gRPC service receives the request, it passes the image to either the TensorFlow or TFlite Interpreter to classify the image and returns the result. The result is then sent back as a gRPC response including classification probabilities.

### 4.7. Containerized gRPC Service

The containerized gRPC service is a version of the gRPC service that is containerized as a Docker container (see Figure 8). Docker containers provide an executable, lightweight, and standalone container image that encapsulates everything the gRPC service needs in order to run. This service image is deployed in Google Cloud using the Cloud Run platform. Containerized gRPC service reduces efforts in deploying gRPC service into the cloud especially when they are already supported by the cloud platform, such as the Google Cloud Run platform that have been used here. Cloud Run provides a fully managed serverless platform to deploy highly scalable containerized applications. The containerized gRPC service could not replace the gRPC service as Docker containers do not have full support for many of the AI libraries for different processor architectures such as armv7 and aarch64 in Raspberry Pi and Jetson. Therefore, offering this variety of technologies and software platform allows services to be instantiated anywhere in cloud, fog, and edge layers.

### 4.8. Diagnosis Request Service

The diagnosis request service has been developed using Android studio, so that it could run on Android devices. This service is responsible for image selection and communication with various diagnosis services. Algorithm 4 shows the procedure that the diagnosis request service follows to get a skin diagnosis prediction from one of the skin image diagnosis services and present the final result.

**Figure 13:**
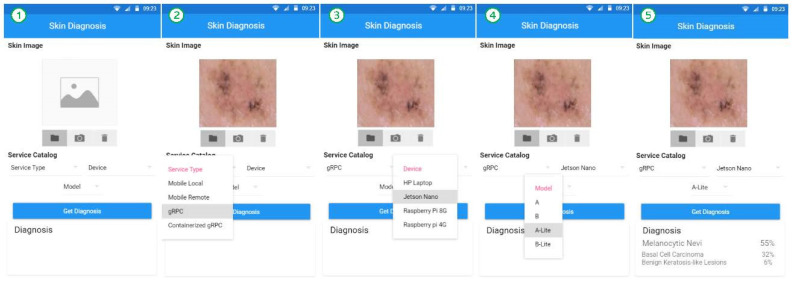
Mobile application user interface for access to skin diagnosis services.

The algorithm takes, as an input, the user selected skin image and the chosen service type from the provided service catalog. In the case of mobile local service, the local service installed in the device will be used for skin image classification directly. In other cases, the diagnosis request service first establishes a connection with the required service. If the mobile remote service is chosen, the application listens to the nearby service broadcasts and establishes a connection with a nearby mobile device. For gRPC-based services (gRPC and containerized gRPC), the application uses gRPC stubs to communicate with the services. When the connection is ready, the diagnosis request is sent along with the skin image to be classified (diagnosed) by the chosen diagnosis service and when the results are sent back, they are presented to the user. Figure 13 shows screenshots of the user interface for the skin diagnosis application, which enables the user to request a diagnosis service. The screenshots are numbered from 1 to 5 to show the steps involved in selecting a service and obtaining a diagnosis on the application.
**Algorithm 4**: Diagnosis_Request_Service**Input**: skin_image, service_type **Output**: class of skin disease1   Function: skin_diagnosis (skin_image, service_type) 2    Init: ip ← grpc server ip address, crt ← server certificate, url ← cloud service URL, 3       service_id ← nearby service ID, user_name ← given device name 4    Try 5       If service_type = = mobile_local_service Then           //request from the local service 6         P[p_0_, … ,p_C_] ← mobile_local_service.get_diagnosis (skin_image) 7       Else If service_type = = mobile_remote_service Then           //Create connection with the nearby device 8           connection ← request_connection(user_name)           //Send a request to the diagnosis service 9           P[p_0_, … ,p_C_] ← connection.get_diagnosis (skin_image) 10      Else If service_type = = grpc_service Then             //Create a secure channel with the diagnosis service 11         channel ← create_secure_channel (ip, crt) 12         P[p_0_, … ,p_C_] ← channel.get_diagnosis (skin_image) 13      Else If service_type = = containerized_grpc_service Then             //Create a secure channel with the cloud 14         channel ← create_secure_channel (url) 15         P[p_0_, … ,p_C_] ← channel.get_diagnosis (skin_image) 16      End If          //Find the largest probability value 17      probability ← p_0_ 18      prediction_class ← 0 19      For c in C 20         If P[c] > probability Then 21             probability ← P[c] 22             prediction_class ← c 23         End If 24      End For          //Find corresponding labels 25      prediction_label ← get_prediction_label(prediction_class) 26      Return prediction_label 27   Catch exception 28      Return error_message 29   End Try

## 5. Service Evaluation and Analysis

This section presents and discusses our experiments and results. First, the experiment settings are presented (Section 5.1). Then, every evaluation metric has been discussed and evaluated, including processing time (Section 5.2), response time (Section 5.3), network time (Section 5.4), service data transfer rate (Section 5.5), and the services’ energy consumption (Section 5.6) and values (Section 5.7).

### 5.1. Experimental Settings

The experiments were conducted in a real-life environment in a typical family home setting to represent everyday city life. They took place over a period of several weeks. Every week, they were conducted for four consecutive days (from Saturday to Tuesday), at three different times of the day. Unfortunately, limited human, and other, resources, made it impossible to conduct the experiments more frequently (every three h) and for the seven weekdays. Table 7 lists the various evaluation variables for which data had been collected during the experiments and those were recorded as testing logs. The table lists the variable names, definitions, units, and an example of collected data.

**Figure 14:**
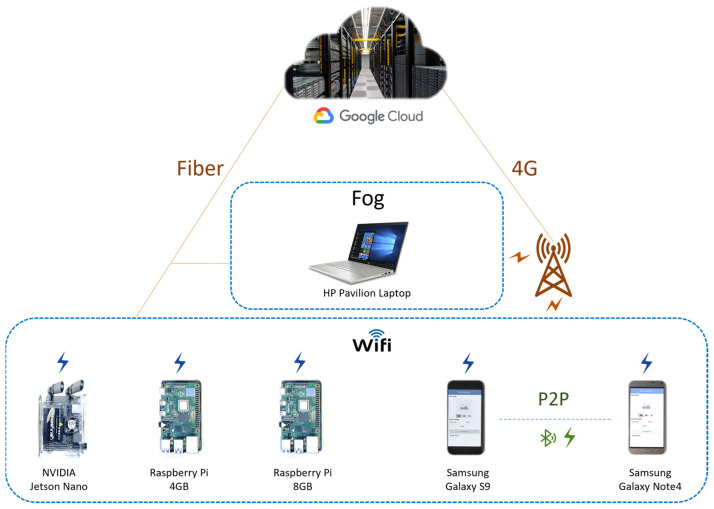
Networking setup.

Figure 14 shows the networking setup for the experiments. All edge devices are connected to a WiFi router that provides a local connection between them and a connection to the Cloud through the Fiber and 4G networks. Two WiFi routers have been used separately for the two different experiment settings. One is the Fiber WiFi router which is both a fiber optic modem and WiFi router that is connected to the fiber optic cable provided by Internet Service Provider (ISP). The second is a 4G WiFi router connected to the 4G cellular network via a SIM card provided by ISP. The smartphones use the Android Nearby Connections API to create a peer-to-peer (P2P) connection between them, which uses either WiFi or Bluetooth for communication. The figure shows the Fog node connects to the edge WiFi network through the 4G and Fiber networks. This is depicted to show how it should be connected in reality and to avoid confusion for the reader. However, the Fog device in our case is connected to the edge devices through the same two routers. This is done due to the human and infrastructure resource limitations since having the fog node in a separate network requires a separate physical space and human support for conducting experiments. In our case, this is an acceptable setup because in studying fog node performance we have focused on the computational performance of the fog node which depends on the device compute capability and is virtually independent of the network performance.

### 5.2. Service Processing Time

**Figure 15:**
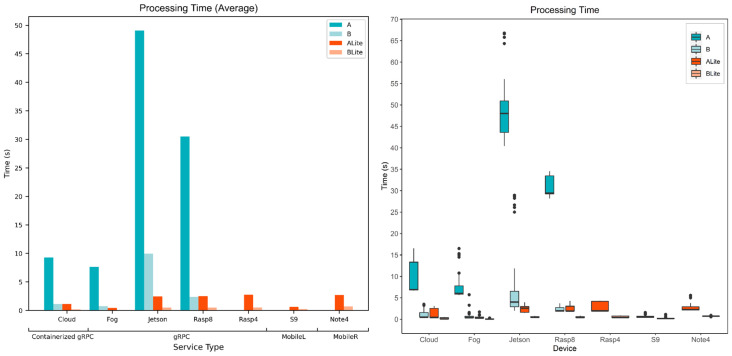
Service processing time (by device).

**Figure 16:**
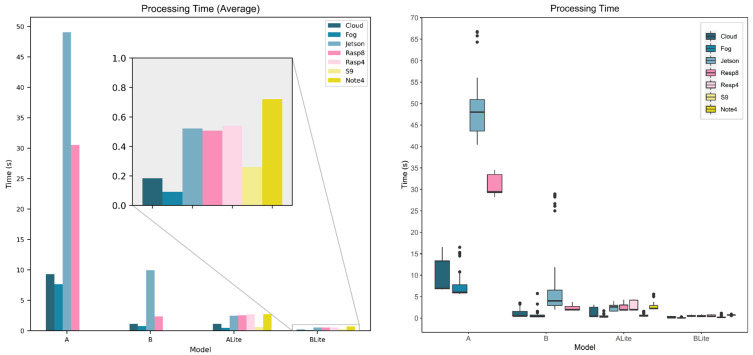
Service processing time (by model).

The processing time is the time that the diagnosis service needs to process an image and predict the skin disease category. It depends on both model complexity and device resources. The processing time was recorded at different times of the day during the week. Figure 15 and Figure 16 show processing times for all service types, devices, and models. Services and devices specifications can be referred to in Table 3.

Figure 15 compares models processing time behavior for each service type and device. The bar chart presents the average processing time where the horizontal axis represents devices, the vertical axis represents the average processing time in seconds, and bars represent model types. For all devices, model A average processing time is higher than that of model B, even for the TFLite versions, which was excepted considering the complexity and size of model A. Jetson device has the highest average processing time for all models compared to other devices and this is related to both Jetson memory limitation and device capability. On Jetson, the average processing times were 49 s, 10 s, 2 s, and 0.5 s for models A, B, ALite, and BLite, respectively. The lowest average processing times were for the Fog device with 7.7 s, 0.8 s, 0.5 s, and 0.1 s for models A, B, ALite, and BLite, respectively.

The boxplot in Figure 15 depicts the processing time data distribution for the whole data collected in our experiments. Boxplots show five statistical measurements the minimum, first quartile (Q1), median (Q2), third quartile (Q3), and maximum. The first quartile (Q1) is the 25th percentile, the median (Q2) is the 50th percentile, and the third quartile (Q3) is the 75th percentile of data. These are depicted as the bottom line in the colored box, the middle thick line inside, and the top line of the box, respectively. The distance between Q1 and Q3 (the height of the colored box) is called the interquartile range (IQR). The maximum and minimum values are the highest and lowest points of the vertical lines on the top and the bottom of the colored boxes. They are calculated using the quartiles and IQR, Q3 + 1.5 × IQR for the maximum and Q1 − 1.5 × IQR for the minimum. Any value more than maximum or less than minimum values is considered as an outlier and depicted as a circle outside the boxplot. Jetson minimum, Q1, Q2, Q3, and maximum processing times for model A were 41 s, 43 s, 48 s, 51 s, and 55 s, respectively, with some outliers over 65 s (see Figure 15 boxplot). This shows that all recorded values for Jetson model A are greater than any other devices or models. Model B processing times distribution on Jetson was much better with 13 s as the maximum value though there were some outliers over 25 s.

Figure 16 compares the device’s processing time behavior for each model. The bar chart presents the average processing time where the horizontal axis represents models, the vertical axis represents the average processing time in seconds, and the bars represent the seven devices that were being evaluated. The Fog had the lowest average processing times among all devices for all models and this can be related to the resources of the fog device. An HP Pavilion Laptop had been used here as a fog device which has an Intel^®^ Core™ i7-8550U processer and 8 GB memory. The Fog has, even, outperformed the Cloud average processing time, 9.3 s, 1.1 s, 1.15 s, and 0.18 s (Cloud) compared to 7.7 s, 0.8 s, 0.5 s, and 0.1 s (Fog) for models A, B, ALite, and BLite, respectively. It seems that the vCPU assigned by Google for the containerized gRPC service is less powerful than the Intel^®^ Core™ i7-8550U processer in the Fog device (gRPC service). The exact physical CPU the containerized gRPC service run on is unspecified by Google on the Google Cloud Run platform.

For models A and B, the Cloud provided the second-best average processing time (9.3 s and 1.1 s), followed by Rasp8 (30.5 s and 2.4 s), and Jetson (49.1 s and 9.9 s), respectively. Rasp8 and Rasp4 average processing times were almost identical for TFLite models (around 3 s ALite and 0.5 s BLite), though Rasp4 could not process original TF models due to memory shortage. For the ALite model, after the Fog (0.46 s), the S9 was the fastest (0.61 s) followed by the Cloud (1.15 s), Jetson (2.48 s), Rasp8 (2.52 s), Note 4 (2.72 s), and Rasp4 (2.74 s). For the BLite model, after the Fog (0.09 s), the Cloud was the fastest (0.18 s), followed by S9 (0.26 s), Jetson (0.52 s), Rasp8 (0.51 s), Rasp4 (0.53 s), and Note 4 (0.72 s). Although both S9 and Note 4 are mobile devices, S9 showed better results due to its processor capabilities (see Table 3).

Looking at previous observations, the processing time is closely related to both device capabilities and the model size and complexity. TFLite optimization greatly improved the processing time and there was no accuracy loss (in our case). In more complex models, the accuracy may lower to a certain level, which may jeopardize the application, depending on its criticality. Devices at the fog or edge layers showed acceptable results compared to the cloud which make them great candidates for local processing.

### 5.3. Service Response Time

**Figure 17:**
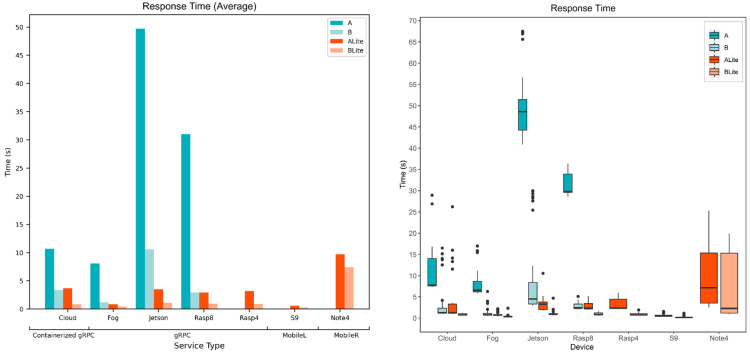
Service response time (by device).

**Figure 18:**
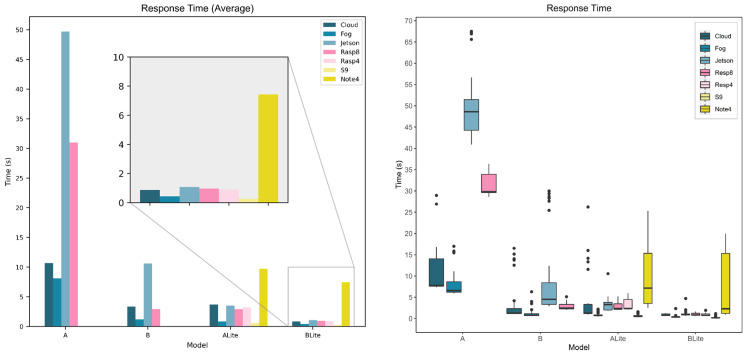
Service response time (by model).

The response time is the total time since the request was made until the result is returned; this includes the processing time. Figure 17 and Figure 18 show the response time of all service types, devices, and models. Service and device specifications are referred to in Table 3. Figure 17 compares the models’ response time behavior for each service type and device. The bar chart presents the average response time where the horizontal axis represents devices, the vertical axis represents the average response time in seconds, and the bars represent model types. The average processing time (see Figure 15) and the average response time (Figure 17) have similar behavior. Model A average response time is always more than model B’s average response time for both TF and TFLite versions. Unlike other devices, the Cloud performed better for model B (3.36 s) than model ALite (3.7 s), and Rasp8 had an identical average response time (2.9 s) for both model B and ALite. Looking at the boxplot in Figure 17 which shows the data distribution of the response time for all the data collected in the experiments. There is a difference in the Cloud performance distribution of model B and ALite. Model ALite IQR is larger than the IQR of model B though both medians (Q2) are at around 1 s. CloudALite had an outliner above 25 s, while the highest CloudB outliner is at around 17 s. For Rasp8, both model B and ALite had a similar distribution, although the maximum value of ALite is more than that of B.

Figure 18 compares the devices’ response time behavior for each model. The bar chart presents the average response time, where the horizontal axis represents models, the vertical axis represents the average response time in seconds, and the bars represent the seven devices that were being evaluated. Although the Cloud and Fog had a close average processing time (see Figure 16), the difference between them is greater when it comes to response times. The difference between the Cloud and Fog average processing times is 1.6 s, 0.4 s, 0.7 s, and 0 s for models A, B, ALite, and BLite, respectively; the difference between their average response times is 2.6 s, 2.2 s, 2.8 s, and 0.4 s for models A, B, ALite, and BLite, respectively. The Cloud average response time is higher as it requires more time to transfer the image across the internet. The networking time metric that is covered in the next section clearly shows the load on the network of different services. The Jetson average processing time greatly affects its response time, especially for model A (48.7 s), with more than 18 s difference with the next highest response time, which is for Rasp8 (31 s). S9 had the lowest average response times for TFLite models 0.6 s (ALite) and 0.3 s (BLite), while Note 4 had the highest average response time among TFLite models (10 s and 7 s), which can be related to the Android Nearby Connections API that was used for mobile device services.

The boxplot in Figure 18 provides a deeper look at the service’s response time values showing the distribution of all collected response times. Note 4 boxplots for ALite and BLite show that the maximum response times were 25 s and 20 s while the medians (Q2) were 7 s and 2 s. BLite median is close to the Q3 (1 s), which means that 50% of collected response times for Note 4 was below 1 s. This variation of data means that this type of service response time is highly unpredictable.

These results show that local processing at mobile devices with MobileL services had the best response time as they do not require any network communication, though only TFLite or small models can be accommodated. On the other hand, other fog and edge devices at the local network such as the Fog and Rasp8 can accommodate more complex models and provide fast responses to local service requests.

### 5.4. Service Network Time

The network time is calculated as the difference between the response time and the processing time, so it includes connection initialization time, devices network processing, and data transfer time (see Equation (4)). This metric shows both the load on the network for each service type and the performance of the different network connection technologies used to communicate with the diagnosis services. There are two basic types of communication protocols that have been used in this research, namely, the nearby connections and gRPC. The mobile remote services use the Android Nearby Connections API to create a peer-to-peer connection using various technologies such as Bluetooth, Wi-Fi, IP, and audio depending on the available connection. Other services use gRPC and SSL for communication and the mobile local service does not require any network communication. In addition, the network time gives an indication of the data transfer factor of the total response time.

(4) NetworkTime=ResponseTime− ServiceProcessTime

#### 5.4.1. Model and Device Behavior

**Figure 19:**
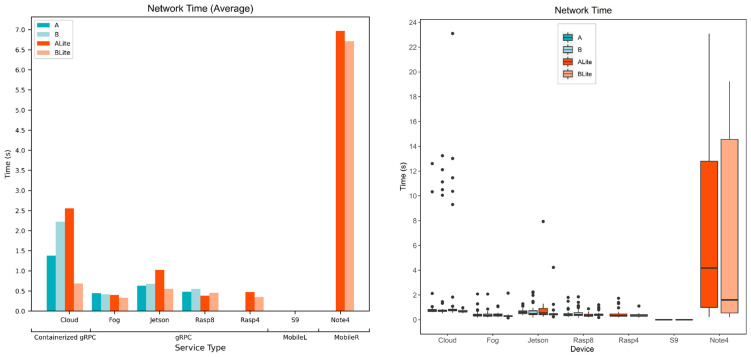
Service network time (by device).

**Figure 20:**
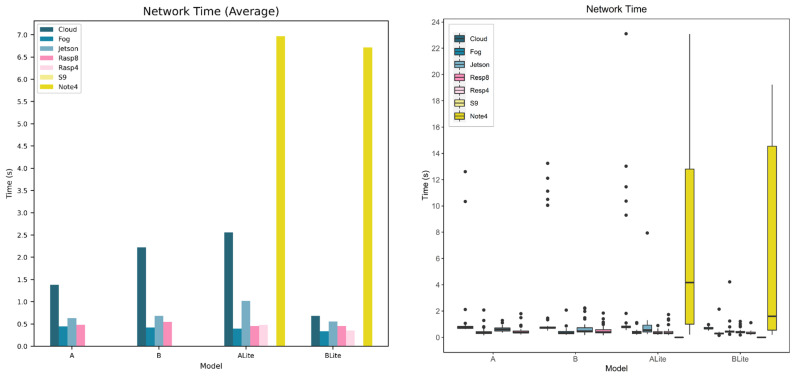
Service network time (by model).

In this section, the network time is evaluated for various devices and models. Figure 19 and Figure 20 show the calculated network time values from the collected response and processing times data. Figure 19 focuses on models’ behavior for each service type and device while Figure 20 focuses on the behavior of the devices for each model.

The bar chart on the left hand side of Figure 19 presents the average network time for all service types and devices in terms of model types. MobileR services had the highest values of average network time (around 7 s), and this can be related to the nearby connections, especially since the network time includes the time required for connection initialization. The difference of average network time between different models on the same device is very small for all devices except the Cloud. The average network time of CloudBLite was 0.6 s, while for other models the network time was 1.4 s, 2.2 s, and 2.6 s (for A, B, and ALite, respectively). However, looking at the right-hand side of Figure 19, the network time boxplot shows the distribution of the data. The Q1, Q2, and Q3 of the Cloud network time for all models fall below 1 s, though there are a lot of anomalies above 9 s for models A, B, and BLite, which have affected the average value. All other devices have a similar distribution of network time but with fewer and much lower anomalies, except for the mobile remote. The maximum value of MobileRALite is 23 s and MobileRBLite is 19 s, both have a minimum of 0.5 s. MobileRALite median is 4 s and MobileRBLite is median 2 s. This high variation of network time on MobileR indicates that the nearby connections are more unpredictable.

Figure 20 compares calculated network time in terms of devices for each model. The bar chart on the left side shows the average network time. Despite the MobileR, whose behavior was explained earlier, the Cloud had the highest values among them. This is expected, as it is the only service that is located across WAN, and all other services are on LAN. Among devices on the LAN, the Fog had the best average network time for all models of around 0.39 s. Resp (Rasp4 0.41 s and Rasp8 0.46 s) was the second best followed by Jetson (0.72 s). The boxplot on the right side shows the distribution of these values. The Cloud had the highest anomalies followed by Jetson. All other device network times fall below 2 s, including all anomalies.

To summarize, the results confirm that both the type of connection and technique used for communication are affecting the networking time. Local services are always the best option if the available resources are sufficient for processing although the available network cards and other device specifications showed a variation of network times among devices on the same LAN.

#### 5.4.2. Behavior over Weekdays

**Figure 21:**
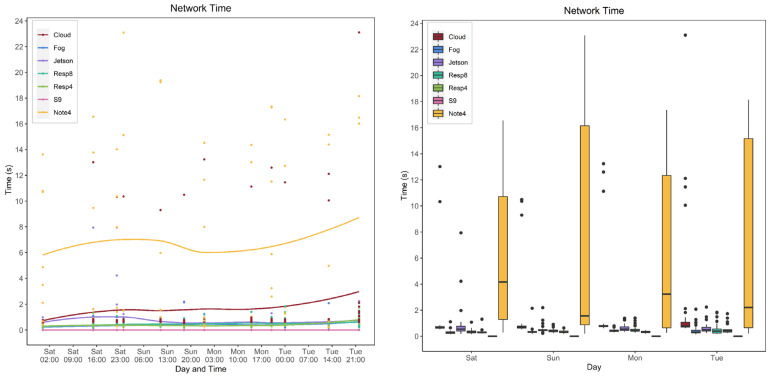
Network time at different times and days.

This section describes the network time, which has been evaluated over the whole period of the experiment to investigate the behavior of the devices. The data were collected for four days starting from Saturday to Tuesday, three times a day for all models and devices. Figure 21 shows the calculated network times plotted over a time series. In the scatter plot on the left side of Figure 21, the network times were plotted as colored dots where each color represents a different device. The vertical axis represents the network time in seconds, the horizontal axis represents the time series including days and hours, the dots represent the calculated network time for each device at a specific time, and the line shows the trend of the network time over time. The trend curve is plotted using the LOESS (Locally Estimated Scatterplot Smoothing) regression analysis method. The S9 mobile device has no network time as it runs a mobile local service that does not require any network communication.

The highest network time trend line (top line) is for Note 4, which runs a remote mobile service. The behavior of the Nearby Connections API has been observed in the previous section, which had a very high distribution of data (see the boxplot of Note 4 in Figure 19). Similarly, it can be seen that the Note 4 data points are spread all over the graph, with a maximum of 23 s on Sunday 00:00 and a minimum of around 0.1 s on Tuesday 13:00. The Cloud is the second-worst network time (second trend line from the top). However, there are eleven values over the Note 4 trend line, ten of them ranging from 9 s to 13 s and one 23 s on Tuesday 21:00. Other devices on LAN are showing a similar trend line, except for Jetson (the purple line), which went slightly higher on Saturday until Sunday afternoon. Saturday 15:00 was the highest with 8 s, and the second highest was on Saturday 22:00 with around 4 s network time. The Fog, Rasp8, and Rasp4 were more stable with one point over the Cloud trend line for the Fog at around 2 s on Sunday 18:00.

Looking at the shape of the trend lines over time, all the lines were lower on Sunday 00:00 and higher on Tuesday 22:00. Note 4 trend line fluctuated more than the other lines, the curve rose on Saturday afternoon until Sunday night. On Monday at daytime, the network time was lower, and then the curve started to rise again from Monday evening until the end of the period. The Cloud network time trend started with a low network time at 1 s on Saturday 00:00, then started to build up, and stabilized at around 2 s from Saturday evening to Monday evening, before it rose again from Monday night to Tuesday night reaching 3 s.

The boxplot on the right side of Figure 21 shows the distribution of calculated network times on different days for different devices. Due to the space limitation in the figure, only the distribution of days has been plotted, not specific times. The boxplot confirms the earlier observation made from the scatter plot. The large boxes of Note 4 confirm the high distribution of network times on the days shown in the scatter plot. Similarly, the Cloud had many outliers over the maximum values on all days and the high outliers for Jetson on Saturday confirm the curve in the Jetson trend line.

To summarize, the results showed there are changes in the device’s network times on different times and days. These changes could be related to the user’s network usage trend at different times of the day and during weekends and weekdays. Further investigation is needed to find trends in network usage. Such information could be used for network and service placement planning which could improve the QoS.

#### 5.4.3. Cellular (4G) vs. Fiber Networks

In this section, a comparative study is made of fiber-optic and cellular 4G internet connections. An experiment has been conducted over three days, from Sunday 28 March 2021 to Tuesday 31 March 2021. The data were collected for both fiber and 4G at two different times of the day, and both Internet connections were from the same network provider. The Cloud services are the ones that require the internet connection to connect to them as they were installed in the Google datacenter. All other services do not require an internet connection as they were installed in the LAN.

**Figure 22:**
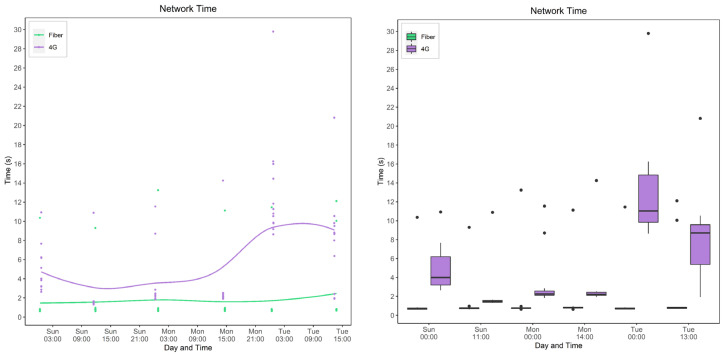
Network time for fiber and 4G of the cloud services.

Figure 22 shows the network time of all Cloud services (for all models) for both fiber and 4G Internet connections. The vertical axis represents the network time in seconds and the horizontal axis represents the time series including days and hours. In the scatter plot on the left side of Figure 22, the dots represent the calculated network time for each connection at a specific time, and the line shows the trend of the network time over time. The trend curve is plotted using the LOESS regression analysis method. As expected, the fiber connection had a better network time (around 2 s) than 4G (ranging from 3 s to 10 s). The fiber connection is more stable over time with a slight rise at the end of the period to around 2.5 s. However, there are a few (seven points total) higher values between 9 s and 13 s. The cellular (4G) connection is less stable over time as the trend line fluctuates over time with many high and low values. The lowest value was 1.5 s on Sunday 28 March 2021 at 11:00, and the highest value was 30 s on Tuesday 31 March 2021 at 00:00. It appears that there was higher demand on the cellular network from Monday night to Tuesday afternoon and lower demand on Sunday afternoon to Monday afternoon, which produced these variations.

The boxplot on the right side of Figure 22 shows the distribution of calculated network times for both fiber and 4G internet connections over time. The boxplot confirms our earlier observation from the scatter plot. The large boxes of the 4G network on Sunday 00:00, Tuesday 00:00, and Tuesday 13:00 are aligned with the curve in the 4G trend line in the scatter plot. The fiber network was much more stable, with smaller IQRs, consistent medians, and few outliners over the whole period.

### 5.5. Service Data Transfer Rate

**Figure 23:**
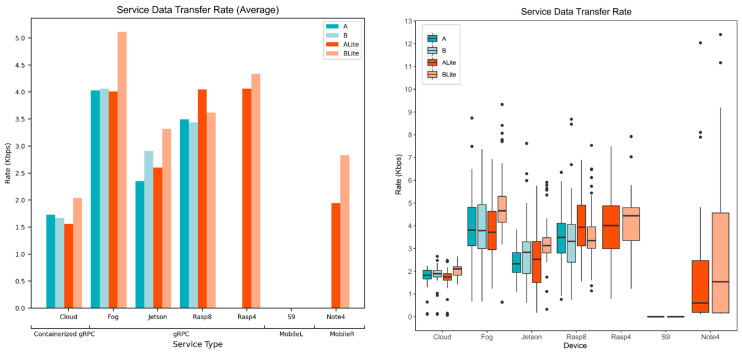
Service data transfer rate (by device).

**Figure 24:**
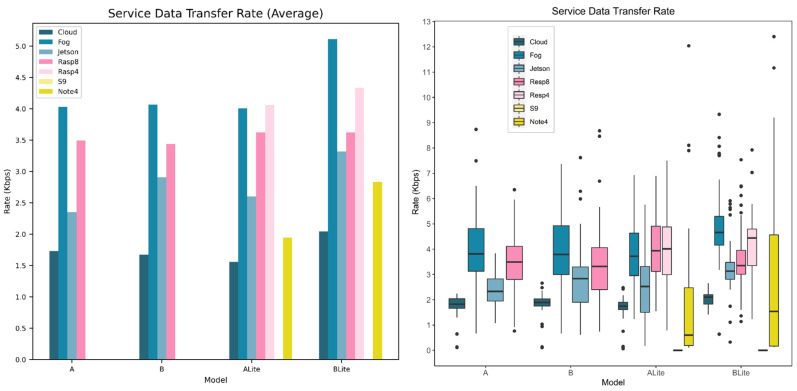
Service data transfer rate (by model).

The service data transfer rate metric is the rate at which the data are being transferred from the request service to the diagnosis service and back again. It includes the time needed for the operating system to initialize the connection, prepare the packets, and send them across the network. Mobile local services do not have a service data transfer rate, as they do not require network communications. The service data transfer rate is calculated as the total size of the transferred data divided by the network time (see Equation (5)). The RequestSize and the ResponseSize are sizes of the request and response packets in bits. Figure 23 and Figure 24 show the calculated service data transfer rate from the collected packet sizes and calculated network times.

(5) $$\mathrm{ServiceDataTransfrerRate}\;=\frac{\mathrm{ResponseSize}+\;\mathrm{RequestSize}\;}{\mathrm{NetworkTime}}$$

Figure 23 compares the service data transfer rate of different models for each service type and device. The bar chart presents the average service data transfer rate where the horizontal axis represents devices, the vertical axis represents the average service data transfer rate in Kbps, and bars represent model types. The gRPC service on the Fog device had the highest average service data transfer rate for all models 4 Kbps for A, B, and ALite as well as 5 Kbps for BLite, which is aligned with the average network time discussed earlier. The Cloud service had the lowest service data transfer rate among all models, 1.7 Kbps, 1.7 Kbps, 1.6 Kbps, and 2 Kbps for models A, B, ALite, and BLite, respectively. This was expected, as the Cloud services are the only services that require the data to be transferred across WAN. The boxplot on the right side of Figure 23 shows the distribution of the service data transfer rates. All devices show larger boxplots than the Cloud’s boxplots, this means that the service data transfer rate for all local devices varies in its values more than the Cloud’s values. Note 4 showed a very low service data transfer rate, with minimum and Q1 values of around 0.1 Kbps. In addition, the medians of both MobileRALite (0.6 Kbps) and MobileRBLite (1.5 Kbps) are lower than those of CloudALite (1.9 Kbps) and CloudBLite (2.1 Kbps).

Figure 24 compares the service data transfer rate of different devices for each model type. For all original TF models, the Fog had the best data service transfer rate followed by Resp8, Jetson, and Cloud. For the ALite model, Rasp4 was better than the Fog by 0.06 Kbps, and they were followed by Resp8, Jetson, Note 4, and Cloud. For the BLite model, the Fog was the best followed by Rasp4, Resp8, Jetson, Note 4, and Cloud. The boxplot on the right side of Figure 24 shows the distribution of the service data transfer rates. The medians of the original TF models show the same pattern as the average values; however, the TFLite models showed a slightly different pattern. Unlike the averages, Note 4 medians were lower than the Clouds, and Rasp4 and Rasp8 both had a similar median of 4 Kbps for ALite model.

### 5.6. Service Energy Consumption

**Figure 25:**
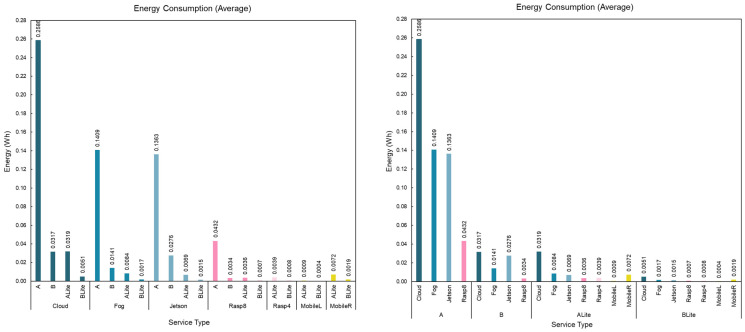
Service energy consumption.

Energy is a key factor for system efficiency in terms of cost and environmental sustainability. Therefore, services that consume less energy are favorable. Figure 25 shows the estimated average energy consumption per task for all service types presented in service catalog (see Table 3). The bar chart on the left side shows energy consumption grouped in terms of devices, while the one on the right side shows energy consumption grouped in terms of models. MobileL had the lowest energy consumption for both ALite (0.0009 Wh) and BLite (0.0004 Wh), as no energy is used on data transfer in those models. The Cloud had the highest energy consumption for all models, 0.26 Wh, 0.03 Wh, 0.03 Wh, and 0.01 Wh for models A, B, ALite, and BLite, respectively. The BLite model consumed the least energy for all service types, compared to other models which was expected, considering the characteristics of this model. On the other hand, model A had the highest energy consumption due to its computation and memory requirements. The CloudA had the highest energy consumption of 0.26 Wh followed by FogA (0.14 Wh), JetsonA (0.14 Wh), and Rasp8 A (0.04 Wh).

### 5.7. Service Value (eValue and sValue)

Two relative values are calculated, one for energy (eValue) and the other for speed (sValue) (see Section 3.5). These service values are used to compare the 22 different service types in terms of their accuracy, energy, and speed (response time). We only used the Fiber network in these calculations (the same applies to the energy consumption values presented in the previous section). The service values are computed using appropriate energy consumption parameters (see Section 3.5). For example, the Cloud eValue uses both Fiber and Wi-Fi energy consumption values. For Bluetooth, in the figures, we used the same energy consumption as for the Wi-Fi but this could easily be replaced by precise Bluetooth energy values. Note that there are also no problems in computing and plotting service values for the 4G network, but this will lengthen the paper and unnecessarily add to its complexity. The comparison provided for 4G versus Fiber in Section 5.4.3 only presents a comparison between network times; all other values, such as the service values, can be drawn from it. This is to bring another design dimension to the reader’s attention, while keeping the article complexity to a minimum.

**Figure 26:**
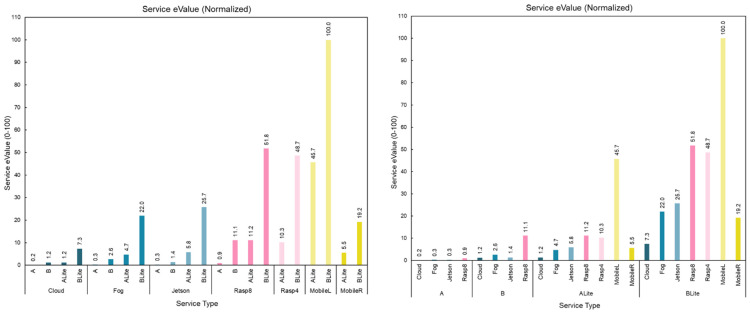
Service eValue.

Figure 26 shows normalized service eValues as an integer between 0 and 100 for all service types. The bar chart on the left side shows the service eValues grouped in terms of devices, while the one on the right side shows the service eValues grouped in terms of models. MobileLBLite had the highest service eValue, and CloudA had the lowest eValue, which is aligned with their energy consumption. In general, the BLite model had the highest values among other models, and model A had the lowest values. When it comes to devices, MobileL services had the best service eValues, though they can only run TFLite models. MobileL services do not require network communication, which eliminates the network data transfer energy from the energy equation (see Equation (1)), reduces their energy consumption, and increases their eValues. The Rasp8 services had the best service eValue among services that run original TF models, and they are the second best for TFLite models after MobileL. This can be related to the energy consumption of the Raspberry Pi devices, which is the lowest among all devices used in the experiments (see Table 3). The Cloud services had the worst eValues due to both devices and data transfer energy consumptions.

**Figure 27:**
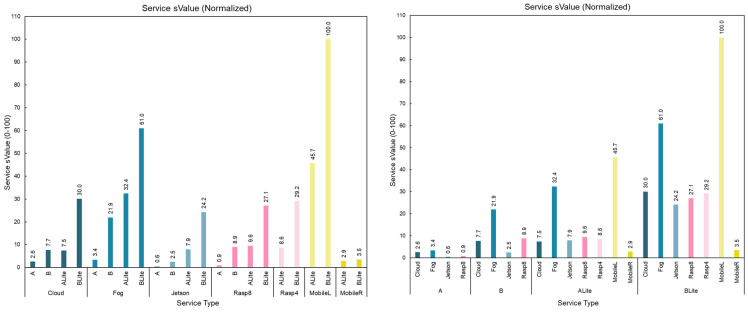
Service sValue.

Figure 27 shows normalized service sValues as an integer between 0 and 100 for all service types. The bar chart on the left side shows the service sValues grouped in terms of devices, while the one on the right side shows the service sValues grouped in terms of models. MobileLBLite had the highest service eValue, and JetsonA had the lowest sValue. For devices running TFLite models, MobileR had the lowest sValues, and for devices running TF models, Jetson had the lowest sValues. In general, MobileL had the best sValues, and the Fog services came in second place. Rasp8 and Rasp4 had similar sValues, and the Cloud services’ were better than those for A and BLite models. The sValue is strongly related to the services’ response times, which have been discussed extensively in Section 5.3.

To summarize, MobileL services had the highest eValue and sValue, as they are using less energy and provide faster responses. The only concern with MobileL services is that they are limited in their resources and cannot accommodate large and complex models or large volumes of data. The Cloud services were much better in terms of sValues but not eValues due to their high energy consumption. The Fog also performed very well in terms of sValues (they are the second-best), but Rasp8 outperformed them when it came to eValues. Jetson services had closer eValue and sValue, as their high processing time affected both energy and response time.

## 6. Conclusions and Future Work

Digital services are the fundamental building blocks of technology-driven smart cities and societies. There has been an increasing need for distributed services that provide intelligence near the fog and edge for reasons such as privacy, security, performance, and costs. The healthcare sector is not an exception; not only does it require such distributed services, but also it is also driven by many other factors including declining public health, increase in chronic diseases, ageing population, rising healthcare costs, and COVID-19.

In this paper, the Imtidad reference architecture is proposed, implemented, and evaluated. It provides DAIaaS over the cloud, fog, and edge using a service catalog case study containing 22 AI skin disease diagnosis services. These services belong to four service classes that are distinguished by software platforms (containerized gRPC, etc.) and are executed on a range of hardware platforms (NVIDIA Jetson nano, etc.) and four network types (Fiber, etc.). The AI models for diagnosis included two standard and two Tiny AI Deep Neural Networks to enable their execution at the edge. They were trained and tested using 10,015 real-life dermatoscopic images.

A detailed evaluation of the DAIaaS skin lesion diagnosis services was provided using several benchmarks. A DL service on a local smartphone provides the best service in terms of energy followed by a Raspberry Pi edge device. A DL service on a local smartphone provides the best service also in terms of speed followed by a laptop device in the fog layer. DL services in the edge layer on local smartphones are the best in terms of energy and response time (speed) as they do not require any network communication, though they can only accommodate TFLite or small models. TFLite optimization provided a great improvement in terms of processing time and compatibility with edge devices. However, it could reduce model accuracy to some levels that could be tolerated depending on the criticality of the application and user preferences. Therefore, we considered the accuracy of the model in both eValue and sValue, to provide a way for the user to choose and trade-off between these factors, energy, and speed. Other devices in the fog and edge layers, such as a laptop and Raspberry Pi (8 GB), can accommodate more complex models and at the same time provide fast responses to local service requests. DL service on a remote smartphone provided unpredictable behavior in terms of network time compared to other edge and fog services due to the Android Nearby Connections API, which is used for nearby smartphone communication. The Cloud services’ processing time is close to the Fog services, though the response time is higher as it requires more time to transfer the image across the internet. This would depend on particular scenarios, such as those requiring heavy computations, which would render the cloud to have much faster responses because in those cases the processing time would be a bottleneck for low-resource fog devices. DL services in the cloud layer also depend on the type of internet connection used. Our evaluation of both Fiber and Cellular (4G) internet connections on the Cloud services confirmed that the fiber network connection is more stable and has lower network time than the cellular connection (4G in this case, but this may change for 5G and 6G). Obviously, while fiber connection was shown to be more stable, it has limitations in terms of user mobility. The Cloud services eValue and sValue are both affected by the required network communication over WAN.

The novelty and the high impact of this research lies in the developed reference architecture, the service catalog offering a large number of services, the potential for the implementation of innovative use cases through the edge, fog, and cloud, and their evaluation on many software, hardware, and networking platforms, as well as a detailed description of the architecture and case study. To the best of the authors’ knowledge, this is the first research paper in which a reference architecture for DAIaaS is proposed and implemented, as well as in which a healthcare application (skin lesion diagnosis) is developed and studied in detail. This work is expected to have an extensive impact on developing smart distributed service infrastructures for healthcare and other sectors.

Future research on distributed services will focus on improving the accuracy and other performance aspects of the skin disease AI model and services. While the design, implementation, and evaluation of the proposed reference architecture and DAIaaS services is detailed and diverse, human, computer, and network resource limitations impeded a higher diversity of hardware, networks, and more frequent measurements. Future lines of research will be oriented towards improving the granularity of the measurements as well as adding to the diversity of the software, hardware, and communication platforms.

Future work will also consider improving and refining the reference architecture, extending it through the development of services in other application domains and sectors including many smart city applications that we have developed over the years including smart cities [2,3,81], big data [8,20], improving computing algorithms [82,83], education [1], spam detection [84], accident and disaster management [85,86], autonomous vehicles and transportation [87,88,89,90,91], and healthcare [6,56,92,93].

AI will be an important parameter in the evolution of the 5th Generation (5G) networks and the conceptualization and design of the 6th Generation (6G) networks. Technologies such as network function virtualization (NFV), software-defined networking (SDN), 3D network architectures, and energy harvesting strategies will play important roles in delivering the promises of 5G and 6G networks. However, it is AI that is expected to be the main player in network design and operations, not only in terms of the use of AI for the optimization of network functions, but also due to the expectations that AI, being a fundamental ingredient of smart applications, will be a major workload to be supported by next-generation networks. While 5G promises us high-speed mobile internet, 6G pledges to support ubiquitous AI services through next-generation softwarization, heterogeneity, and configurability of networks [13]. The work on 6G is in its infancy and requires the community to conceptualize and develop its design, implementation, deployment, and use cases [13]. This paper is part of our broader work on distributed AI as a Service and is a timely contribution to this area of developing next-generation infrastructure, including the network infrastructure, needed to support smart societies of the future. Our earlier work [13] proposed a framework for provisioning Distributed AI as a service in IoE (Internet of Everything) and 6G environments and evaluated it using three case studies on distributed AI as service delivery in smart environments, including a smart airport and a smart district. This paper adds to the earlier work by extending another case study on developing a service catalog of distributed services.

## Figures and Tables

**Table 1:** Related works: Tiny AI at the edge.

Reference	Application Domain	Application	AI Model
Zebin et al. [47]	Monitoring and Healthcare Systems	Human Activity Recognition	Custom CNN Model
Benhamida et al. [48]	Autonomous Vehicles	Traffic Sign Recognition	SSD MobileNetV2
Zeroual et al. [50]	Security (Authentication)	Face Recognition	VggNET
Alsing [49]	Smart Homes	Object (Notes) Detection	R-CNN, SSD, and Tiny YOLO
Soltani et al. [52]	Wireless Networking	Signal Modulation Classification	DeepSig CNN
Domozi et al. [53]	UAVs Search and Rescue	Object Detection	SSD
Ahmadi et al. [51]	Security	Malware Detection	IntelliAV (Random Forests)

**Table 2:** Related works: skin disease diagnosis.

Reference	AI	Approach	Classes	Datasets
Jha et al. [63]	Double U-Net	Segmentation	7	MICCAI 2015, CVC-ClinicDB, ISIC-2018, and Science Bowl 2018
Bajwa et al. [64]	CNN Ensemble	Classification	7–23	ISIC 2018 and DermNet
Zhang et al. [65]	CNN	Classification	4	Clinical dataset Peking Union Medical College Hospital
Wei et al. [66]	CNN Ensemble	Classification	7	ISIC 2018
Liu et al. [67]	CNN on multi-image, demographic information and medical history	Classification	27	Collected from teledermatology practice in U.S.
Gavrilov et al. [68]	CNN	Classification	2	ISIC 10,000 expanded to 1,000,000 using distortions
Garcia et al. [72]	Fuzzy algorithm	Segmentation	3	ISIC 2016 and ISIC 2017

**Table 3:** The Imtidad service catalog.

	Service Type	Layers	Platform	Platform Specifications	Platform Energy	Network Specification	Latency	Model
1	Containerized gRPC Service	Cloud or Fog	Google Cloud Compute Node	2 CPUs 8 GB Memory 80 Concurrency	100 W [75]	Fiber or Cellular	Depends on Network	A
2								B
3								ALite
4								BLite
5	gRPC Service	Fog or edge	HP Pavilion Laptop	CPU: Intel^®^ Core™ i7-8550U @ 1.80 GHz (Turbo up to 4.00 Ghz) 8 GB Memory	Idle: 10.2 W Working: 66.3 W	Wireless LAN Frequency Band: 2.4 GHz/5 GHz Data rate < 450 Mbps	Partially Configurable	A
6								B
7								ALite
8								BLite
9		Fog or Edge	NVIDIA Jetson nano	GPU: 128-core Maxwell CPU: Quad-core ARM A57 @ 1.43 GHz 4 GB Memory	5–10 W	Wireless LAN Frequency Band: 2.4 GHz/5 GHz Data rate < 450 Mbps	Partially Configurable	A
10								B
11								ALite
12								BLite
13		Fog or Edge	Raspberry Pi Model B (8 GB)	1.5 GHz Quad-core ARM Cortex-A72 8 GB Memory 2.4/5.0 GHz IEEE 802.11ac wireless	Idle: 2.7 W Working: 5.1 W	Wireless LAN Frequency Band: 2.4 GHz/5 GHz Data rate < 450 Mbps	Partially Configurable	A
14								B
15								ALite
16								BLite
17		Fog or Edge	Raspberry Pi Model B (4 GB)	1.5 GHz Quad-core ARM Cortex-A72 4 GB Memory 2.4/5.0 GHz IEEE 802.11ac wireless	Idle: 2.7 W Working: 5.1 W	Wireless LAN Frequency Band: 2.4 GHz/5 GHz Data rate < 450 Mbps	Partially Configurable	ALite
18								BLite
19	Mobile Local Service	Edge	Samsung Galaxy S9	GPU: ARM Mali-G72 MP18 Octa-Core, 2 CPUs: 2.7 Ghz Quad-Core Mongoose M3 1.8 Ghz Quad-Core ARM Cortex-A55 4 GB Memory	Idle: 1.09 W Working: 5.16 W	Wireless LAN Frequency Band: 2.4 GHz/5 GHz Data rate < 450 Mbps	Partially Configurable	ALite
20								BLite
21	Mobile Remote Service	Edge	Samsung Galaxy Note 4	GPU: ARM Mali-T760 MP6 Octa-Core, 2 CPUs: 1.9 GHz Quad-core ARM Cortex-A57 1.3 GHz Quad-core ARM Cortex-A53 4 GB Memory	Idle: 1.4 W Working: 9.4 W	Wireless LAN Frequency Band: 2.4 GHz/5 GHz Data rate < 450 Mbps	Partially Configurable	ALite
22								BLite

**Table 4:** The acronyms used for the services and service definitions.

	Acronym	Definition
1	CloudA	Model A (Executed on Cloud)
2	CloudB	Model B (Executed on Cloud)
3	CouldALite	Model ALite (Executed on Cloud)
4	CloudBLite	Model BLite (Executed on Cloud)
5	FogA	Model A (Executed on Fog—HP Laptop)
6	FogB	Model B (Executed on Fog—HP Laptop)
7	FogALite	Model ALite (Executed on Fog—HP Laptop)
8	FogBLite	Model BLite (Executed on Fog—HP Laptop)
9	JetsonA	Model A (Executed on Jetson)
10	JetsonB	Model B (Executed on Jetson)
11	JetsonALite	Model ALite (Executed on Jetson)
12	JetsonBLite	Model BLite (Executed on Jetson)
13	Rasp8A	Model A (Executed on Raspberry pi 8 GB)
14	Rasp8B	Model B (Executed on Raspberry pi 8 GB)
15	Rasp8ALite	Model ALite (Executed on Raspberry pi 8 GB)
16	Rasp8BLite	Model BLite (Executed on Raspberry pi 8 GB)
17	Rasp4ALite	Model ALite (Executed on Raspberry pi 4 GB)
18	Rasp4BLite	Model BLite (Executed on Raspberry pi 4 GB)
19	MobileLALite	Model ALite (Executed on Local Mobile—Galaxy S9)
20	MobileLBLite	Model BLite (Executed on Local Mobile—Galaxy S9)
21	MobileRALite	Model ALite (Executed on Remote Mobile—Galaxy Note 4)
22	MobileRBLite	Model BLite (Executed on Remote Mobile—Galaxy Note 4)

**Table 5:** HAM10000 dataset characteristics.

Class	Diagnostic Categories	Code	Images	Sample
0	Actinic Keratoses and Intraepithelial Carcinoma/Bowen’s Disease	akiec	327	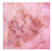
1	Basal Cell Carcinoma	bcc	514	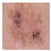
2	Benign Keratosis-Like Lesions (Solar Lentigines/Seborrheic Keratoses and Lichen-Planus-Like Keratoses)	bkl	1099	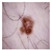
3	Dermatofibroma	df	115	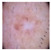
4	Melanoma	mel	1113	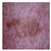
5	Melanocytic Nevi	nv	6705	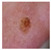
6	Vascular Lesions (Angiomas, Angiokeratomas, Pyogenic Granulomas, and Hemorrhage)	vasc	142	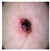
Total Number of Images			10,015	

**Table 6:** Comparison between original TF and TFLite Models.

Model	Layers	Total Parameters	TensorFlow				TensorFlow Lite		Size Ratio
			RAM (MB)	Training (%)	Validation (%)	Testing (%)	RAM (MB)	Testing (%)	
A	55	23,057,255	264	95.64	83.23	82.38	87.8	82.38	3.01
B	19	3,833,367	43.9	79.05	78.28	77.33	14.6	77.33	

**Table 7:** Evaluation data variables and examples.

Variable	Definition	Unit	Example
Date	Date of the request	Date	16/02/2021
Day	Day of the week	Date	Tuesday
Hour	Time of the request	Time	06:15:49.333
ImageName	Image name from the dataset	String	ISIC_0033458
ModelVersion	Model used for classification (A = 1, B = 2, ALite = 3, BLite = 4)	Number	1
DeviceName	The name of the device: Cloud, Laptop, Jetson, Rasp8, Rasp4, S9, Note 4	String	Cloud
RequestSize	The packet size of the request message	Bytes	171,316
RequestSentTimestamp	The timestamp when the request is sent by the user	Milliseconds	1,613,445,211,566
RequestReceiveTimestamp	The timestamp when the request is received at the service	Milliseconds	1,613,445,342,877
ServiceProcessTime	The service processing time (inference or compute)	Milliseconds	6334
ResponseSentTimestamp	The timestamp when the response is sent from the service	Milliseconds	1,613,445,349,211
ResponseReceiveTimestamp	The timestamp when the response is received at the user device	Milliseconds	1,613,445,349,325
ResponseTime	The total time taken to obtain a response, from the moment the request had been made	Milliseconds	137,759
ResponseSize	The packet size of the response message	Bytes	112
Result	Diagnosis results as a probability of each class of the diseases (7 values for 7 classes separated by commas)	List of floats	[4.025427 × 10^−7^, 1.1340192 × 10^−7^, 4.8968374 × 10^−8^, 6.5097774 × 10^−6^, 3.8256036 × 10^−7^, 0.00020890325, 0.9997837]

## Data Availability

The HAM10000 dataset is a public dataset available from the link provided in the article.
